# Bulge-Forming miRNases Cleave Oncogenic miRNAs at the Central Loop Region in a Sequence-Specific Manner

**DOI:** 10.3390/ijms23126562

**Published:** 2022-06-12

**Authors:** Olga Patutina, Daria Chiglintseva, Bahareh Amirloo, David Clarke, Svetlana Gaponova, Valentin Vlassov, Elena Bichenkova, Marina Zenkova

**Affiliations:** 1 Institute of Chemical Biology and Fundamental Medicine SB RAS, Lavrentiev’s Ave. 8, 630090 Novosibirsk, Russia; patutina@niboch.nsc.ru (O.P.); dashachiglintseva@gmail.com (D.C.); sveta-mira@yandex.ru (S.G.); vvv@niboch.nsc.ru (V.V.); 2 Faculty of Biology, Medicine and Health, School of Health Sciences, University of Manchester, Oxford Rd, Manchester M13 9PT, UK; bahareh.amirloo@manchester.ac.uk (B.A.); david.clarke@manchester.ac.uk (D.C.); elena.v.bichenkova@manchester.ac.uk (E.B.)

**Keywords:** microRNA, miRNases, peptide–oligonucleotide conjugates, artificial ribonucleases, hybridization, RNA cleavage, turnover, RNase H

## Abstract

The selective degradation of disease-associated microRNA is promising for the development of new therapeutic approaches. In this study, we engineered a series of bulge-loop-forming oligonucleotides conjugated with catalytic peptide [(LeuArg)_2_Gly]_2_ (BC–miRNases) capable of recognizing and destroying oncogenic miR-17 and miR-21. The principle behind the design of BC–miRNase is the cleavage of miRNA at a three-nucleotide bulge loop that forms in the central loop region, which is essential for the biological competence of miRNA. A thorough study of mono- and bis-BC–miRNases (containing one or two catalytic peptides, respectively) revealed that: (i) the sequence of miRNA bulge loops and neighbouring motifs are of fundamental importance for efficient miRNA cleavage (i.e., motifs containing repeating pyrimidine–A bonds are more susceptible to cleavage); (ii) the incorporation of the second catalytic peptide in the same molecular scaffold increases the potency of BC–miRNase, providing a complete degradation of miR-17 within 72 h; (iii) the synergetic co-operation of BC–miRNases with RNase H accelerates the rate of miRNA catalytic cleavage by both the conjugate and the enzyme. Such synergy allows the rapid destruction of constantly emerging miRNA to maintain sufficient knockdown and achieve a desired therapeutic effect.

## 1. Introduction

Abnormal gene expression has been recognized as one of the key hallmarks of various types of human pathologies. Small non-coding RNAs, microRNAs, which act mainly as negative regulators of gene expression, orchestrate gene expression profiles through binding to complementary sequences in the 3′ untranslated region (UTR) of target mRNAs, thus guiding their translational repression or complete degradation [1,2]. MicroRNAs (miRNAs) have therefore emerged as key players in the development of many human diseases, including cancer [3,4], autoimmune disorders [5], and neurodegenerative [2,6,7] and cardiovascular diseases [8].

Given the strong involvement of abnormally expressed miRNAs in the initiation and development of various pathological states in humans, the selective knockdown of specific regulatory miRNAs can potentially reduce or even reverse disease progression. Approaches developed for the sequence-specific inactivation of abnormally expressed miRNAs include: (i) miRNA-masking antisense oligonucleotides [9,10] designed to block the access of miRNAs to a targeted mRNA via direct binding to its 3′ UTR region through Watson–Crick base-paring; (ii) synthetic anti-miRNA antisense oligonucleotides (anti-miRs) or their chemically modified analogues forming stable, fully complementary complexes with mature miRNA [11,12,13]; (iii) miRNA mopping-up approaches, such as miRNA sponges [14,15], which contain multiple miRNA recognition sites, and miRNA zippers [16] capable of hybridizing in tandem with the 3′ and 5′ ends of two individual miRNAs. Since most of the mentioned approaches are based on the tight binding of miRNA with the oligonucleotide scaffold, some of them can also activate cellular RNase H to trigger irreversible miRNA cleavage, in addition to a steric hindrance. This is why alterations of the sugar-phosphate backbone introduced for their protection against cellular nucleases compatible with RNase H recruitment are advantageous [12,17].

However, the biggest challenge is that some human miRNAs are present in much greater copy numbers per cell (1000–30,000) [18,19] in comparison to mRNAs (typically, fewer than 100 per cell) [19]. Recent studies have shown that miRNAs rank among the most rapidly produced and long-lived cellular RNAs. Indeed, the rate of miRNA production (for instance, 110 ± 50 copies/cell/min for miR-21) is approximately ten times higher than the rate of mRNA generation in a cell, and the median half-life for Ago2-bound miRNA reaches 25 h versus 2.2 h for mRNAs [20,21]. More rapid change in the mRNA profile in response to environmental changes maintains the highly adaptive properties of cells, while the persistence of miRNAs provides regulatory stability. Such stability, manifested in high intracellular concentrations (up to 22 µM) [19] and survivability, make miRNA a challenging target for suppression. Thus, in order to achieve sufficient downregulation of pathogenic miRNAs and gain a desirable therapeutic effect, multiple turnover catalysis is critical for irreversibly inactivating many copies of continuously emerging miRNA in a persistent way.

Considering all the approaches mentioned above, one of the most promising for the downregulation of overexpressed pathogenic miRNAs could be sequence-specific artificial ribonucleases (aRNases), which are conjugates of antisense oligonucleotides and chemical moieties exhibiting ribonuclease activity and triggering the multi-turnover catalytic cleavage of RNA targets [22]. A number of recent studies have shown that such sequence-specific aRNases can efficiently execute the highly selective inhibition of disease-associated RNAs in vitro and in vivo [23,24,25,26,27,28]. In particular, we developed miRNA-specific artificial ribonucleases, “miRNases”, the structural scaffold of which incorporates a hairpin antisense oligonucleotide and a catalytic peptide. The engineered miRNases were capable of selectively recognizing and destroying highly oncogenic miR-21, showing substrate turnover and—what is especially significant—acting synergistically with RNase H. The developed miRNases were shown to be highly effective inhibitors due to their ability to irreversibly degrade miRNA in a catalytic mode, thus leading to remarkable therapeutic efficiency, as manifested in apoptosis induction in cancer cells, the suppression of cell invasiveness, and the inhibition of cell proliferation in vitro and tumour growth in vivo [24,25].

The most successful “hairpin” miRNases attacked mainly the terminal region located at the 3′ end of the target miR-21 (bases 15–21) because the catalytic peptide was attached to the 5′ end of the recognition motif [24]. To increase catalytic turnover, it may be preferable to cleave the target miRNA in its central part. In this case, due to the shortening of formed miRNA fragments, the cleavage products may dissociate much more readily from the complex with miRNase. Moreover, this strategy contributes to a complete loss of normal functioning of miRNA molecules. The “dual” miRNases developed earlier, with the catalytic peptide embedded between two short miRNA-specific recognition motifs via long linkers, represented the first attempt to achieve cleavage in the central part of the miRNA sequence [28]. However, the ability of miRNA sequences to form relatively stable secondary structures and hairpins [29] requires more forceful strand invasion strategies to unfold them and expose this functionally significant region for successful degradation.

Over the past two decades, many data have been accumulated showing an enhanced sensitivity in RNA bulge loops to cleavage by various agents [30,31,32,33,34,35,36,37]. The efficiency of bulge-loop cleavage by metal-independent peptide aRNases has not been studied until recently [38,39]. The latest research shows the exceptional importance of the spatial positioning of the catalytic peptide in aRNase structure with respect to phosphodiester bonds in the targeted RNA bulge loop. The catalytic peptide should be attached through an aminohexyl linker located in the α- or β-configuration at the C1′ position of abasic deoxyribose, which provides much more flexibility [38]. It was also revealed that doubling the number of catalytic peptides in a peptide–oligonucleotide conjugate significantly accelerates the rate of target cleavage [39].

In this study, the developed concept was applied to design bulge-forming peptide–oligonucleotide conjugates, BC–miRNases, for cleaving therapeutically significant targets—microRNAs. RNA cleavage in the bulge loops induced by various aRNases has been extensively investigated for model RNAs (short synthetic RNA substrates, yeast tRNA^Phe^) [30,32,33,34,38,39]; however, the sensitivity of RNA bulges induced in natural molecules therapeutically relevant to aRNases has not been investigated so far. Herein, a series of bulge-forming miRNases presumably displaying enhanced cleavage efficiency were engineered to target highly oncogenic miR-17 and miR-21 in a three-nucleotide bulge loop induced in the miRNA central loop region upon binding with BC–miRNases. The designed BC–miRNAses consist of a recognition oligonucleotide bearing in its central part one or two catalytic peptides attached to the C1′ position of an abasic deoxyribose residue and located opposite the bulge loop. The comprehensive study of the biochemical characteristics of BC–miRNases included an assessment of their hybridization properties, a comparative analysis of the catalytic activity of mono- and bis-peptide conjugates, as well as the definition of their nucleotide base specificity. Furthermore, our research illuminates the future prospects of designed RNases as miRNA inhibitors under intracellular conditions implying RNase H activity.

## 2. Results

### 2.1. Design and Synthesis of Bulge-Forming miRNases

MiRNA-specific bulge-loop-forming ribonucleases, BC–miRNases, were designed for this study to selectively target two oncogenic miRNAs, miR-21-5p and miR-17-5p (hereafter referred to as miR-21 and miR-17, respectively), the overexpression of which is an integral characteristic of malignant growth [40,41,42]. The series of synthetic ribonucleases was generated through the covalent attachment of one or two catalytic peptides to an antisense oligonucleotide capable of selectively binding a miRNA target (i.e., either miR-21 or miR-17) and forming a 3 nt bulge loop in the central part of miRNA upon hybridization for subsequent cleavage. Such a loop size was previously found to be sufficient for effective cleavage without compromising efficient hybridization between bulge-loop-inducing conjugates and RNA targets [38]. As a part of the design process, we calculated the thermodynamic parameters (ΔG, ΔH, and ΔS) and T_m_ values for each potential complex between the recognition oligonucleotide and the corresponding miRNA (miR-17 or miR-21) using the DINAMelt Server (www.unafold.org (accessed on 11 March 2022)). Some representative structures from this analysis, along with the corresponding T_m_, ΔG, ΔH, and ΔS values, are shown in the Supplementary Materials, Figure S1. The fully complementary oligonucleotides formed highly stable duplexes with both miR-17 and miR-21 (Figure S1a,d, respectively). The formation of the 3 nt bulge loop upon the hybridization of miR-17 with the bulge-forming oligonucleotides miR-17- ON-α and miR-17-ON-β destabilized the duplex, leading to a decrease in T_m_ by almost 15 °C compared to the “perfect match” (Figure S1a,b). An even higher level of destabilization was detected in the case of miR-21 hybridization with miR-21-ON-α1, miR-21-ON-β1, miR-21-ON-α2, and miR-21-ON-β2 (Figure S1e,f), when T_m_ values decreased by nearly 19 °C compared to the corresponding “perfect match”. However, the insertion of the additional abasic deoxyribose residue (dR) into the recognition motif of the bis-oligonucleotides miR-17-ON-αα and miR-17-ON-ββ considerably improved the hybridization power of these oligonucleotides, presumably by relaxing the induced conformational tension within the bulge-loop region (Figure S1c). Despite a significant decrease in Tm, the designed bulge-forming duplexes were sufficiently stable under physiological conditions.

**Figure 1:**
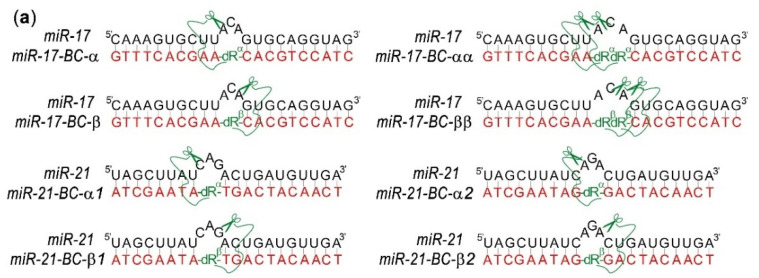

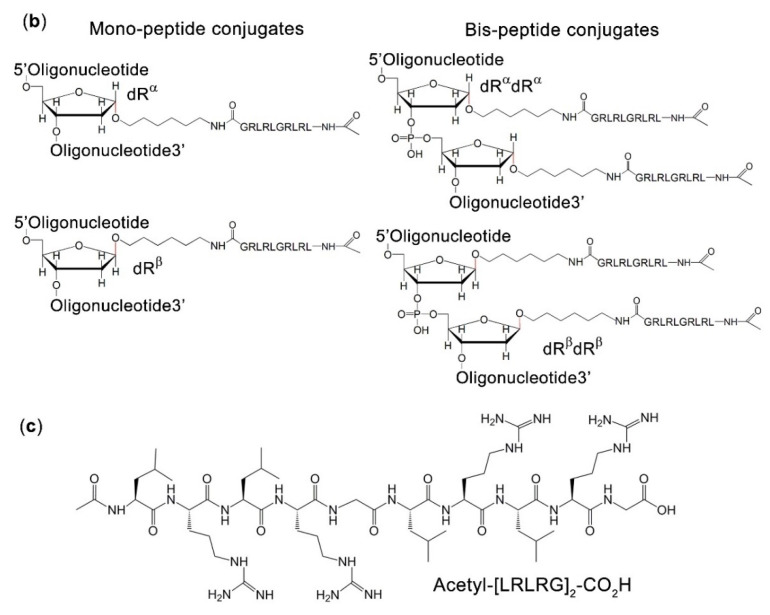
(**a**) Design concept for bulge-forming mono-conjugates (miR-17–BC-α, miR-17–BC-β, miR-21–BC-α1, miR-21–BC-β1, miR-21–BC-α2, and miR-21–BC-β2) and bis-conjugates (miR-17–BC-αα and miR-17–BC-ββ) targeted to miR-17 and miR-21. (**b**) Structural organization of the peptide attachment points within the mono- and bis-peptide–oligonucleotide conjugates, with one or two catalytic peptides Acetyl-[LRLRG]2-CO_2_H coupled to the oligonucleotide recognition motif through aminohexyl linkers attached at the C1′ position of abasic sugar residues in either the α- (dR^α^) or the β-configuration (dR^β^). (**c**) Structure and sequence of the catalytic peptide Acetyl-[LRLRG]_2_-CO_2_H.

The conjugation between the catalytic and recognition structural components of BC–miRNase was achieved through amide coupling between the C-terminal carboxylic group of the Acetyl-[LRLRG]_2_-COOH peptide and the aliphatic amine group of the aminohexyl linker located at the C1′ atom of the internally located abasic nucleotides (dR^α^ or dR^β^) in either an α- or a β-configuration (Figure 1) to explore the cleavage opportunities offered by different orientations of the peptide relative to the induced miRNA bulge loop. Such a design (i) makes the exposed single-stranded RNA region susceptible to transesterification via attacks from closely located catalytic groups [38]; (ii) promotes an ‘in-line’ configuration, which is crucial for catalysis [38,43,44,45,46]; (iii) provides several possible cleavage points within the exposed loop [38,39]; and (iv) diminishes the size of the cleaved RNA products to facilitate the breakdown of the hybridized complex and initiate the next catalytic cycle [39]. Indeed, due to relatively short binding regions, which cover 8–11 nucleotides from the 5′ and 3′ termini of miRNAs, the cleavage of miRNA in the central part may potentially provide a ground for substrate turnover.

Following the above design, we generated a series of eight miRNases, including six mono-peptide conjugates in either the α- or the β-configuration and two bis-peptide conjugates (Figure 1). Two mono-conjugates, miR-17–BC-α and miR-17–BC-β, were designed to target miR-17, whereas the other four mono-conjugates, miR-21–BC-α1, miR-21–BC-β1, miR-21–BC-α2, and miR-21–BC-β2, were targeted to miR-21 and differed in the location of the dR^α^ or dR^β^ relative to the miRNA sequence (Figure 1). In the case of miR-21–BC-α1 and miR-21–BC-β1, the abasic sugar residue was located between nucleotides 8 and 9 of the recognition motif, so that the hybridized miR-21 was forced to form the bulge loop C9–A10–G11. In the case of miR-21–BC-α2 and miR-21–BC-β2, the position of the abasic sugar residue was shifted by one nucleotide, so that the peptide was targeted to the A10–G11–A12 bulge loop induced in miR-21. The latter structural variants were designed to determine the base-specificity of RNA transesterification catalysed by this type of miRNase. Indeed, it was previously shown that the predominance in cleavage of Pyr-A or G-X bonds by peptide–oligonucleotide conjugates is driven by both their structural properties and the sensitivity of these sites to catalytic cleavage [23,24,28]. Two bis-peptide conjugates, miR-17–BC-αα and miR-17–BC-ββ, were deemed to enhance RNA cleavage efficiency through the synchronized actions of two catalytic groups (Figure 1).

The synthesis and full characterization of the conjugates are described in detail in the ‘Materials and Methods’ section. A shift in HPLC retention time from 17.5 min (average for starting oligonucleotides) to 25 min (average for mono-conjugates) and 28.5 min (bisconjugate) was observed, which was reproducible in all conjugation reactions. The identities and purities of the conjugates were confirmed using ^1^H NMR spectroscopy and MALDI–ToF spectrometry (Table 1; Figures S2–S7). The MALDI–ToF mass spectrometric data listed in Table 1 show that the experimental masses of the conjugates were in close agreement with the calculated values.

### 2.2. Hybridization of Bulge-Forming miRNases with miRNA Targets

The hybridization properties of miRNases are essential for providing site-specific cleavage of the target miRNA sequences. Both hybridization and ribonuclease activity of the bulge-loop-forming conjugates were studied in two different buffer systems. Buffer 1 was employed to study RNA cleavage under metal-fee conditions and contained 50 mM Tris–HCl, pH 7.0, 200 mM KCl, and 1 mM EDTA, which is generally used for EDTA-mediated metal ion chelation and for the complete sequestration of any traces of metal ions present in reaction mixtures. Buffer 2 was composed of 20 mM Tris–HCl, pH 7.8, 40 mM KCl, 8 mM MgCl_2_, and 1 mM DTT, which are usually recommended for in vitro reactions with RNase H.

**Figure 2:**
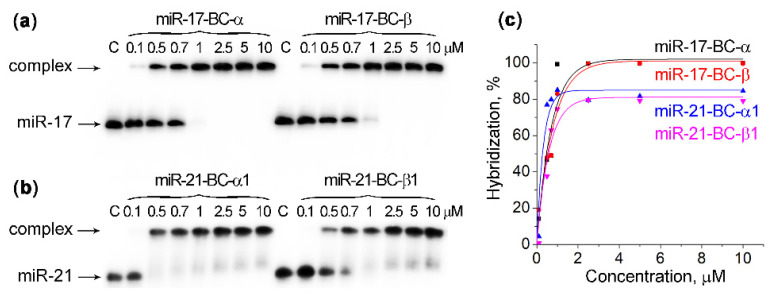
Hybridization of 5′-[^32^P]-miRNAs with BCs in Buffer 1. (**a**,**b**) Radioautographs of 15% native PAAG, showing hybridization of miR-17–BC-α and miR-17–BC-β with miR-17 and miR-21–BC-α1 and miR-21–BC-β1 with miR-21, respectively. miRNAs (1 μM) were incubated with BCs (0.1–10 μM) in Buffer 1 (50 mM Tris–HCl, pH 7.0, 200 mM KCl, and 1 mM EDTA) at 37 °C for 45 min. C: control, miRNA was incubated in the absence of conjugates. The samples were loaded onto the running gel immediately after the reaction was quenched, with 1 min intervals. The concentration (μM) of the conjugate is indicated on the top of electropherograms. (**c**) Concentration profiles of BC hybridization efficiency with miR-17 and miR-21.

Since the hybridization power of the bis-conjugates against RNA sequences was shown to be higher than that of the structurally related mono-conjugates due to additional electrostatic interaction (see Figure S1) [39], the gel-shift experiments and the measurements of K_a_ values were limited to the mono-conjugates miR-17–BC-α, miR-17–BC-β, miR-21–BC-α1, and miR-21–BC-β1. Gel-shift analysis showed that all the studied mono-conjugates exhibited a high affinity with the targets in both buffer systems (Table 1; Figure 2 and Figure S8). For both types of conjugates (i.e., miR-17- and miR-21-specific), the hybridization plateau was reached even at a 1:1 molar ratio of the conjugates to their miRNA targets. In fact, miR-17–BC-α and miR-17–BC-β showed the quantitative binding of miR-17 at concentrations equal to 1 μM or above, thus demonstrating 100% hybridization efficiency. The overall binding efficiency of miR-21–BCs to miR-21 was 85% (Figure 2 and Figure S8). The slightly reduced affinity of conjugates to miR-21 is associated with miRNA sequences (i.e., the GC content of the hybridized region: 35% for miR-21 versus 50% for miR-17). A comparison of the association constants Ka for BCs indicated that the binding efficiency of all BCs in Buffer 1 was approximately 6–10-fold higher than in Buffer 2 (Table 1), probably due to the 0.2 M KCl in Buffer 1 versus the 0.04 M in Buffer 2. In the case of miR-17-targeted BCs, the peptide orientation (either α or β) had no significant effect on the efficiency of BC binding with miRNAs. Indeed, the analysis of the association constants for miR-17–BC-α (Ka = 46.1 ± 15.4 × 10^6^ M^−1^) and miR-17–BC-β (Ka = 42.7 ± 13.4 × 10^6^ M^−1^) showed no statistically significant difference. By contrast, the binding affinity of miR-21–BC-α1 and miR-21–BC-β1 differed significantly (Ka = 96.0 ± 30.0 × 10^6^ M^−1^ and Ka = 4.9 ± 1.7 × 10^6^ M^−1^, respectively), showing that the attachment of the peptide in the α-configuration enhanced the binding affinity to the RNA 20-fold compared to the β-configuration (Table 1). Apparently, the α-configuration of the peptide provides more favourable binding of the conjugates to miR-21.

### 2.3. Efficiency and Specificity of miRNA Cleavage by Mono-Bulge-Loop-Forming miRNases

The assessment of the ribonuclease activity of the bulge-loop-forming miRNases against miR-17 and miR-21 was carried out in a single-turnover mode using a 20-fold molar excess of the conjugate relative to the RNA substrate in order to allow for direct comparisons of their catalytic activities with the previously studied conjugates from the same structural category [38,39]. To maintain identical conditions with those reported earlier [38,39], the target miRNA present at a 1 µM concentration was treated with the corresponding conjugate present at 20 µM for 72 h at 37 °C, and the RNA cleavage products were analyzed using 18% PAGE electrophoresis under denaturing conditions, as described in the Materials and Methods section. The effect of different factors on the ribonuclease activity of the conjugates was studied, including buffer composition, the role of the nucleotide sequence, and the context of the target bulge-loop region, as well as the impact of the secondary structure that the target miRNA sequence can potentially form.

First, we evaluated an inherent sensitivity to a transesterification of the 3 nt bulge loop induced upon the hybridization of miR-17 or miR-21 with the bulge-forming oligonucleotide (miR-17–B-ON or miR-21–B-ON, respectively) lacking the catalytic peptide. In these experiments, 5′-[^32^P]-labelled miRNA was incubated in Buffer 1 or Buffer 2 in the presence of the corresponding B-ON for 72 h (see Figure S9). In Buffer 1, both miR-17 and miR-21 incubated with miR-17–B-ON or miR-21–B-ON, respectively, remained fully intact over the entire period of the incubation. However, when these miRNAs were incubated with the same oligonucleotides in Buffer 2, a minor and rather slow self-cleavage of the bulge-loop regions was witnessed, with overall degradations of 11% and 6% seen for miR-17 and miR-21, respectively (Figure S9). The observed level of miRNA self-cleavage can be attributed to the presence of Mg^2+^ ions in Buffer 2 because Mg^2+^ was previously shown to stimulate the self-cleavage of RNA [47]. These experiments suggest that the hybridization of miRNAs with bulge-loop-forming oligonucleotides may enhance the reactivity of phosphodiester bonds in this single-stranded region, so that a detectable cleavage can be facilitated by metal ions even in the absence of the cleaving groups. We hypothesize here that such enhanced reactivity of the formed bulge loops may be a favourable fact for BC–miRNase-catalysed miRNA cleavage.

**Figure 3:**
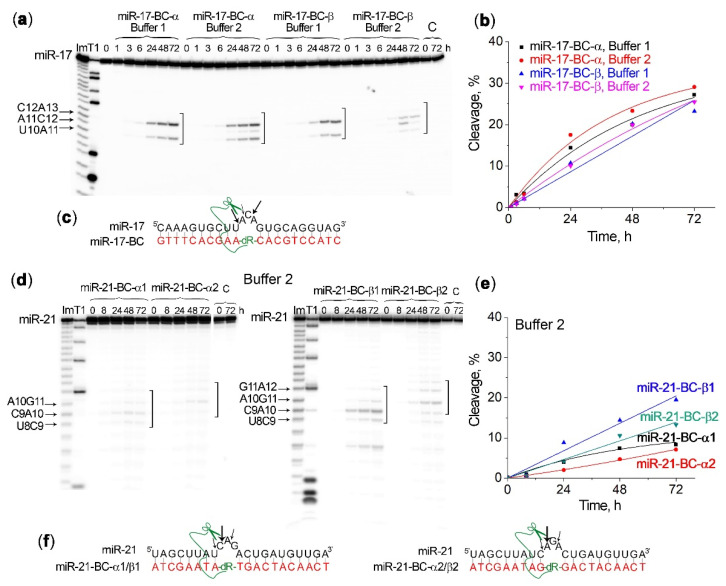
Kinetics of 5′-[^32^P]-miR-17 and 5′-[^32^P]-miR-21 cleavage by mono-conjugates miR-17–BC-α, miR-17–BC-β, miR-21–BC-α1, miR-21–BC-α2, miR-21–BC-β1, and miR-21–BC-β2. (**a**,**d**) Radioautographs of 18% denaturing PAAG showing the cleavage products of miR-17 in Buffer 1 and Buffer 2 and miR-21 in Buffer 2. miRNA (1 µM) and BCs (20 µM) were incubated at 37 °C for 72 h. Lanes Im and T1: imidazole ladder and partial RNA digestion with RNase T1, respectively; C: control, miRNA was incubated in the absence of conjugates. The incubation time (in hours) is shown at the top. The square bracket indicates the bulge-loop region. (**b**,**e**) Progress curves of miR-17 and miR-21 cleavage by BCs. (**c**,**f**) Positions of miR-17 cleavage by miR-17–BC-α/β and miR-21 cleavage by miR-21–BC-α1/β1 and miR-21–BC-α2/β2, respectively. The size of the arrows corresponds to the extent of miRNA cleavage at the particular linkage.

Indeed, the analysis of the ribonuclease activity of the mono-conjugates miR-17–BC-α and miR-17–BC-β against miR-17 showed noticeable, time-dependent cleavage of the loop region (Table 1; Figure 3a,b), which reached 25–30% after 72 h of incubation. The buffer composition did not affect the catalytic performance of these conjugates because neither cleavage extent nor the apparent rate constants (k_obs_) measured for the cleavage reactions in Buffer 1 and Buffer 2 showed statistically significant differences, thus demonstrating that the cleavage of miR-17 was mainly the result of the catalytic activity of the peptide, without reliance upon magnesium ions. Moreover, the ribonuclease activity of the miR-17–BC-α and miR-17–BC-β conjugates seemed to be independent of the α- or β-configuration of the peptide attachment for this series of the conjugates, as both the total cleavage extent and the apparent rate constants (k_obs_) estimated for these two conjugates were practically equal, within the experimental error (Table 1). All three positions within the induced 3 nt bulge loop A11–C12–A13 of miR-17 were cleaved, although with a significant predominance of the C12–A13 site over the U10–A11 site and especially the A11–C12 site. The cleavage extent increased in the row C12–A13 > U10–A11 >> A11–C12 (Figure 3a,c).

Strikingly, miR-21 remained intact in Buffer 1 when treated with the mono-conjugates miR-21–BC-α1, miR-21–BC-β1, miR-21–BC-α2, or 21–BC-β2, although in Buffer 2 some detectable cleavage was observed at the formed bulge-loop region of miR-21 (Figure 3). In the case of miR-21, the β conjugates were more potent compared to their α-counterparts. Indeed, the extent of miR-21 cleavage by miR-21–BC-β1 and miR-21–BC-β2 reached 19% and 13%, respectively, at the 72 h time point (Figure 3d,e; Table 1), whereas the cleavage efficiency of miR-21–BC-α1 and miR-21–BC-α2 was almost twice as low (7–8%) and only slightly exceeded the level of miRNA self-cleavage in a complex with unconjugated oligonucleotide miR-21-ON under these conditions (Figure S9). The apparent rate constants (k_obs_) measured for miR-21–BC-β1 and miR-21–BC-β2 were more than two-fold higher than those seen for the corresponding α-isomers miR-21–BC-α1 and miR-21–BC-α2 (Table 1). The analysis of the miR-21 cleavage profile showed that all three phosphodiester bonds in the formed 3 nt bulge-loop were cleaved by the conjugates; however, as expected, the cleavage patterns differed for miR-21–BC-α1/β1 and miR-21–BC-α2/β2 due to a shift in the induced single-stranded region. The cleavage efficiency of the conjugates miR-21–BC-α1/β1, which cut miR-21 at the C9–A10 bond, was higher than of miR-21–BC-α2/β2, for which the main cleavage site is A10–G11. This finding can be explained by the fact that the C–A bond is one of the most sensitive to cleavage by natural and artificial ribonucleases and also one of the first to undergo self-cleavage [48].

### 2.4. Efficiency and Specificity of miRNA Cleavage by Bis-Bulge-Loop-Forming miRNases

The bis-bulge-loop-inducing conjugates miR-17–BC-αα and miR-17–BC-ββ carried two catalytic peptides attached to two adjacent abasic deoxyribose residues either in αα- or ββ-configuration. The sequence of the oligonucleotide domain in bis-conjugates was the same as in mono-conjugates, which permits a direct comparison of cleavage activity of mono- and bis-miRNases (see Figure 1 for the design).

**Figure 4:**
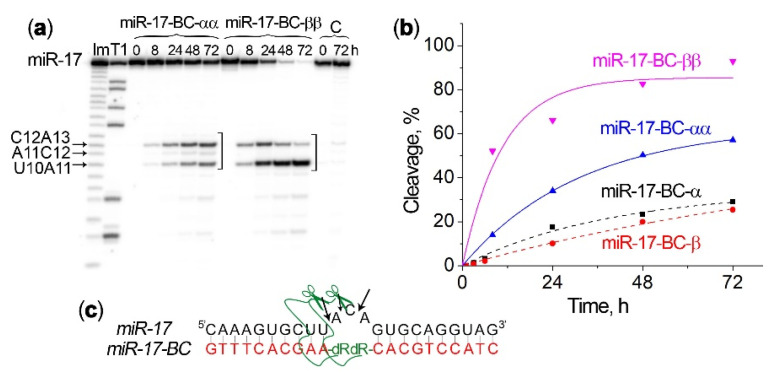
Kinetics of 5′-[^32^P]-miR-17 cleavage by bis-conjugates miR-17–BC-αα and miR-17–BC-ββ. (**a**) Radioautograph of 18% denaturing PAAG showing the cleavage products of miR-17 in Buffer 2. miRNA-17 (1 µM) and BCs (20 µM) were incubated at 37 °C for 72 h. Lanes Im and T1: imidazole ladder and partial RNA digestion with RNase T1, respectively; C: control, miRNA was incubated in the absence of conjugates. The incubation time is shown at the top. The square bracket indicates the bulge-loop region. (**b**) Progress curves of miR-17 cleavage by bis-miR-17–BC-αα and miR-17–BC-ββ. For comparison, the dashed curves show miR-17 cleavage by the mono-peptides miR-17–BC-α and miR-17–BC-β. (**c**) Positions of miR-17 cleavage by miR-17–BC-αα and miR-17–BC-ββ.

The ribonuclease activities of the miR-17–BC-αα and miR-17–BC-ββ conjugates in Buffer 2 were significantly higher than those of miR-17–BC-α and miR-17–BC-β (Figure 4, Table 1). The incorporation of the second peptide in the α-orientation to produce miR-17–BC-αα led to an almost two-fold increase in the level of miR-17 cleavage, from 29.1% to 57.1%, at the 72 h time point (Figure 4b). However, the β-configuration of the extra peptide triggered even greater enhancement of potency. Indeed, the attachment of the second peptide in the β-configuration to produce miR-17–BC-ββ led to a four-fold increase in the level of miR-17 cleavage, thus reaching more than 90% within 48 h and up to 100% of RNA demolition by 72 h (Figure 4b; Table 1). The observed rate constants (k_obs_) for miR-17–BC-αα and miR-17–BC-ββ were two and three times as high, respectively, as those seen for the related mono-conjugates (Table 1).

For miR-17–BC-αα, the main site of miRNA cleavage was C12–A13, with a clear similarity to the cleavage patterns observed for miR-17–BC-α. In the case of miR-17–BC-ββ, during the first 8 h the cleavage at the C12–A13 position also dominated but soon after this the corresponding cleavage product began to disappear, while the cleavage at the U10–A11 position accelerated sharply to make this cleavage site predominant (Figure 4a,c). We assume here that the initially accumulated fragment 5′-[^32^P]-^1^C-^2^A-^3^A-^4^A-^5^G-^6^U-^7^G-^8^C-^9^U-^10^U-^11^A-^12^C-3′ was cleaved further through an additional attack at the U10–A11 bond to generate a shorter cleavage product 5′-[^32^P]-^1^C-^2^A-^3^A-^4^A-^5^G-^6^U-^7^G-^8^C-^9^U-^10^U-3′, which became dominant. The observed results are in line with the data obtained in experiments with tRNA^Phe^, when bulge-forming αα and ββ conjugates promoted a 90% cleavage of tRNA at the bulge loop after 48 h [39].

### 2.5. Efficiency of miRNA Cleavage by Bulge-Forming miRNases in the Presence of RNase H

**Figure 5:**
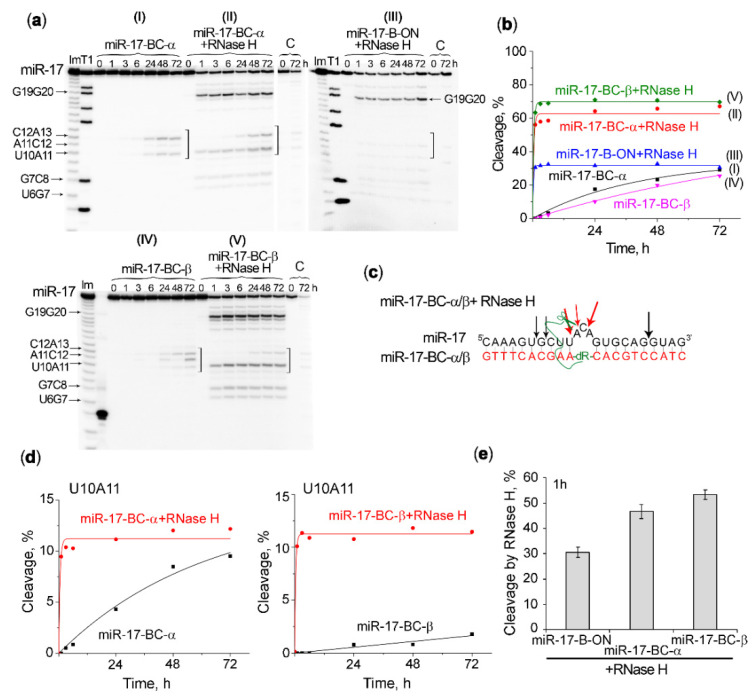
Cleavage of 5′-[^32^P]-miR-17 by miR-17–BC-α, miR-17–BC-β, and/or RNase H. (**a**) Radioautographs of 18% denaturing PAAG showing the profiles of miR-17 cleavage by miR-17–BC-α alone (I), by a combination of miR-17–BC-α and RNase H (II), by RNase H in a complex with the oligonucleotide miR-17–B-ON (III), by miR-17–BC-β alone (IV), or by a combination of miR-17–BC-β and RNase H (V). Duplexes formed between 5′-[^32^P]-miRNA (1 µM) and BC or B-ON (20 µM) were incubated in Buffer 2 at 37 °C for 72 h. RNase H was used at a concentration of 5 u/mL. Lanes Im and T1: imidazole ladder and partial RNA digestion with RNase T1, respectively; C: control, RNA was incubated in the same buffer in the absence of BC or B-ON and in the presence of RNase H. The incubation time is shown at the top. The square bracket indicates the bulge-loop region. (**b**) Progress curves of miR-17 cleavage by BCs and/or RNase H. (**c**) The major positions of miR-17 cleavage by BC (red arrows) and RNase H (black arrows) when acting jointly. (**d**) Progress curves of miR-17 cleavage at the U10–A11 bond by miR-17–BC-α or miR-17–BC-β alone (black) or in combination with RNase H (red). (**e**) The extent of miR-17 cleavage by RNase H in the duplex with ON and in combination with BCs calculated at the 1 h time point (considered only at the cleavage sites inherent in RNase H).

**Figure 6:**
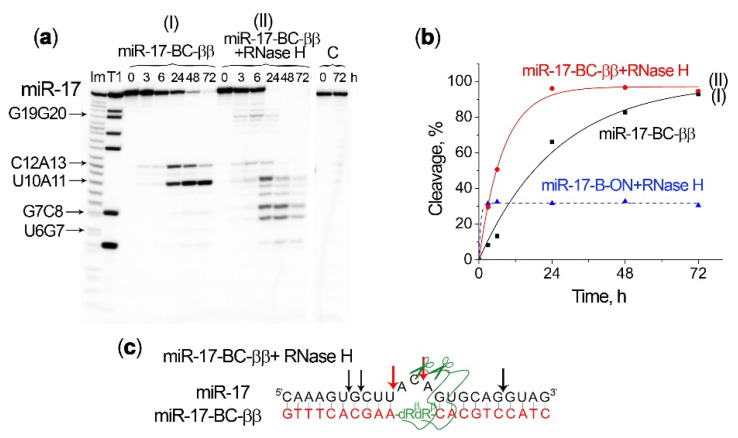
Cleavage of 5′-[^32^P]-miR-17 by miR-17–BC-ββ and/or RNase H. (**a**) Radioautographs of 18% denaturing PAAG showing the profiles of miR-17 cleavage by miR-17–BC-ββ alone (I) and by a combination of miR-17–BC-ββ and RNase H (II). Duplexes formed by 5′-[^32^P]-miRNA (1 µM) and BC (20 µM) were incubated in Buffer 2 at 37 °C for 72 h. RNase H was used at a concentration of 5 u/mL. Lanes Im and T1: imidazole ladder and partial RNA digestion with RNase T1, respectively; C: control, RNA was incubated in the same buffer in the absence of BC and in the presence of RNase H. The incubation time is shown at the top. (**b**) Progress curves of miR-17 cleavage by miR-17–BC-ββ and/or RNase H. For a comparison, the dashed curve shows the cleavage of miR-17 by RNase H in a complex with the oligonucleotide B-ON. (**c**) The major positions of miR-17 cleavage by a combination of miR-17–BC-ββ (red arrows) and RNase H (black arrows).

A major advantage of the conjugates containing unmodified deoxyribooligonucleotide as RNA-recognizing motifs is their ability to recruit RNase H for the cleavage of RNA within the RNA–DNA hybrid, which may significantly increase the efficiency of miRNA degradation [23,25,28]. In order to evaluate the ability of the engineered bulge-forming conjugates to guide RNase H for miRNA cleavage, we compared the kinetics of miR-17 cleavage (1) by one of the mono- or bis-conjugates alone; (2) by RNase H in the absence of the conjugate, when miRNA was pre-hybridized with the unconjugated bulge-forming oligonucleotide; and (3) by the simultaneous action of BC–miRNase and RNase H in the same reaction mixture. The study was performed for the mono-conjugates miR-17–BC-α and miR-17–BC-β (Figure 5) and for the most efficient bis-conjugate miR-17–BC-ββ (Figure 6).

An investigation of the cleavage profile showed that the cleavage of the target by RNase H in the heteroduplex with a complementary oligonucleotide was observed mainly at the 3′ region of miRNA, involving the cuts at the G19–G20 site (Figure 5a(III)). The cleavage curve reaches a plateau after 1 h of incubation, when RNA cleavage reached 30% (Figure 5a(III),b(III)). The simultaneous attack of miRNA by the conjugate and RNase H promoted certain configurational changes in the single-strand–double-strand junction and in the heteroduplex overall, and as a result the primary pattern of miRNA cleavage changed for both the conjugate and RNase H. Under these conditions, the major cleavage site for the conjugates shifted from C12–A13 to U10–A11 (Figure 5a(II,V)). RNase H, by contrast, did not only cleave G19–G20; minor cleavages were also observed at the G7–C8 and U6–G7 linkages in the 5′ region of miRNA. Moreover, the overall level of miR-17 cleavage far outweighed the effects of RNase H and the conjugate when acting separately. The total level of miR-17 cleavage by RNase H and miR-17–BC-α or miR-17–BC-β reached ~70% at the 1 h time point, which was 2.3 times higher than the level of miR-17 cleavage by RNase H in the complex with the oligonucleotide and 20 times higher than the level of miR-17 cleavage by BCs at this time point (Figure 5b), thus demonstrating a synergy between miR-17–BC-α/β and RNase H activity. Indeed, in the presence of RNase H, the cleavage rate of the conjugate increased significantly: the BC-mediated cleavage products were already detected within the first hour of incubation and the extent of cleavage at the U10–A11 bond by BCs increased 10-fold (Figure 5d). The efficiency of miRNA cleavage by RNase H was also enhanced. First, in addition to the cleavage at the G19–G20 site, some modest cleavage at the G7–C8 and U6–G7 bonds was also observed. Second, total miRNA cleavage at the sites typical for RNase H increased 1.5-fold after 1 h of incubation (Figure 5e). It should also be noted that a combination of RNase H and the conjugate in the β-configuration (miR-17–BC-β) led to a higher level of miRNA cleavage than that caused by the combined action of RNase H and miR-17–BC-α. In this case, a more significant mutual enhancement of enzyme efficiency was observed (Figure 5b,d,e), which was probably also associated with the more natural β-configuration of the peptide.

An even more dramatic enhancement in the efficiency and rate of miRNA cleavage was observed with the simultaneous action of RNase H and the bis-conjugate miR-17–BC-ββ (Figure 6a(II)). Indeed, the combined action of miR-17–BC-ββ and RNase H against miR-17 led to a complete degradation of the miRNA target and within a much shorter incubation time (less than 24 h) (Figure 6b). Such a cleavage was accompanied with a rapid accumulation of the shortest cleavage products, corresponding to the cleavage at the G7–C8 and U6–U7 linkages, while all other sensitive sites within the bulge loop were entirely cleaved by this time (Figure 6a(II)). The G7–C8 and U6–G7 sites were typical for RNase H, but as in the case of mono-peptide BCs, these sites were not cleaved when miR-17 was bound to ON-B (Figure 5a(III)) and they became accessible for RNase H only after conjugate-mediated cleavage within the bulge loop took place. Thus, the obtained results confirm the synergistic effect of bulge-forming miRNases and RNase H, which may form the basis for the efficient miRNA-inhibitory activity of BCs in tumour cells.

## 3. Discussion

The main challenge in the sequence-selective destruction of disease-associated miRNA is to achieve a sufficiently high level of its degradation in order to maintain negligible amounts of continuously emerging target molecules. This is exactly the challenge that we confronted here by developing bulge-loop-forming miRNases that offer an opportunity for the multi-turnover knockdown of highly oncogenic miRNAs.

Contrary to the generally accepted theory that all mature miRNAs in the cytoplasm are in the Ago-bound state, it was revealed that miRNAs were present in a 13-fold excess in a stoichiometric relation to Ago2 proteins [49]. Despite the high intracellular concentration of miRNAs, their assembly with Ago2 proteins is a long-term event, which may take up to an hour. A significant fraction of miRNAs are degraded even before reaching Ago2 [50]. Moreover, a certain portion of miRNAs bind to the target mRNA without the mediation of Ago2 and induce mRNA repression at a much later stage by recruiting Ago2 to an already formed heteroduplex [49]. This miRNA relocation behaviour, including the shuttling between Ago2, different target mRNAs, and probably other molecular carriers in the cell, provides potential opportunities for the inhibition of miRNAs by targeting them with miRNases at the transition stages. Considering this, the developed miRNases were designed to target the mature single-stranded form of miRNA localized in the cytoplasm rather than the miRNA precursors that are usually shielded by other proteins and reside at the hairpin state. As was mentioned in the Introduction section, the concept of effective miRNA downregulation was recently demonstrated using mono-peptide “hairpin” miRNases exhibiting a marked biological effect both in cancer cells and in mouse tumour models [23,24,25]. The observed high level of tumour suppression reported for this aRNase (miR-21-miRNase) resulted from both the intrinsic capacity of miRNase to catalyze the sequence-specific cleavage of miR-21 at the 3′-terminal region and the recruitment of RNase H to promote the additional cleavage of other miRNA regions hybridized with the recognition motif of the conjugate [25]. Although the structural design of miRNases aims to eliminate a complete reliance on RNase H activity, it does not exclude the possibility for RNase H recruitment, which may accelerate miRNA cleavage and turnover further by working synergistically with the conjugates.

In the intracellular environment, as well as in the reaction mixture, a majority of miRNAs can adopt a folded (e.g., hairpin) conformation or homodimeric structures, which may potentially represent a barrier to their therapeutic targeting [29]. Figure S10 shows the most stable hairpin structures and possible homodimers for the miR-21 and miR-17 sequences estimated using the OligoAnalyzer software (Integrated DNA Technologies, Inc., Coralville, IA, USA, version 3.1). The central part of both the miR-21 and miR-17 molecules is enclosed in a stable hairpin structure flanked with an extended stem, which may represent a significant obstacle for the cleavage of this region by the catalytic conjugates. In this work, we developed miRNases of a new design that allow for the efficient destruction of miRNA in its central part.

Our study was conducted with the application of synthetic single-stranded miRNA-models the sequences of which completely correspond to the mature miR-17-5p and miR-21-5p presented in murine and human cells. The novel bulge-loop-forming miRNases induced a 3 nt bulge loop in the central part of miRNA upon binding opposite to the attachment point of the catalytic peptide. According to the obtained data (see Figure S9), such a bulge loop is characterized by enhanced reactivity towards catalytic cleavage.

The extent of cleavage in the bulge-loop region (25–30%) of the target miR-17 catalysed by the mono-conjugates miR-17–BC-α and miR-17–BC-β was similar to that observed earlier for “dual” miR-17-targeted conjugates [28] and for the structurally related mono-conjugates BC3-α and BC3-β targeted to the synthetic fluorescently labelled F-Q-RNA BC-conjugates, forming a three-nucleotide bulge loop upon hybridization with the RNA target [39]. On average, the BC3-α–β conjugates were able to reach a 32.9% cleavage in the bulge loops, with BC3-α showing a superiority in cleavage (47.3%) over BC3-β (18.4%) under identical conditions.

Since the catalytic peptide is attached via an aminohexyl linker to the C1′ position of deoxyribose placed in the middle of the recognition oligonucleotide motive, such a design allowed for the incorporation of more than one modified deoxyribose residue in the oligonucleotide and more than one peptide into the conjugate structure to produce bis-conjugates with enhanced potency. Indeed, the attachment of two peptides in an α-orientation doubled the rate of miRNA cleavage (from ~20–25% to 50% within 48 h), while a β-orientation of the peptides achieved a more than four-fold cleavage enhancement (from ~18% to 100% after 72 h of incubation). The obtained data show that the ββ conjugate was significantly more active than its αα analogue, which might be attributed to the fact that, in contrast to αα-BC, ββ-BC has a catalytic peptide replacing the aromatic bases. This may lead to a more advantageous spatial configuration of the peptides in the hybrid complex with RNA, with the β-orientation of the peptides providing a more strategically favourable position against the target sugar–phosphate RNA backbone.

The incorporation of the second peptide fragment into the structures of miR-17–BC-αα and miR-17–BC-ββ enhanced RNA cleavage efficiency by providing additional opportunities for synchronized actions of the catalytic guanidinium groups to form a guanidine–guanidinium dyad in the vicinity of the target phosphodiester bonds, which is critical for the catalysis of RNA transesterification [38,39,51]. Moreover, the addition of the extra abasic nucleotide to the structure of the bis-conjugates improved the hybridization properties of miR-17–BC-αα and miR-17–BC-ββ towards miR-17 (see Figure S1), presumably by alleviating the induced conformational strain within the loop region of the hybridized complex. Moreover, the incorporation of the extra peptide may further reinforce the binding affinity of bis-conjugates with the RNA target through additional electrostatic interactions between the negatively charged RNA chain and the positively charged peptide. Indeed, we previously demonstrated that even one extra peptide, especially when attached in an α-configuration, could enhance the association constants of the bis-conjugates towards the RNA target in a statistically significant manner [39].

The success of the bulge-loop-forming miRNases developed here is manifested in their ability to achieve a full demolition of the hardly accessible central part of the miRNA sequence. The cleavage potential of the best structural variant, miR-17–BC-ββ, was even higher than that offered by the “hairpin” miRNases developed earlier [24,25]. Given that the “hairpin” miR-21-RNase with a single catalytic peptide showed a marked biological effect both in cancer cell lines and in mouse tumour models, the expectations for this new class of bulge-loop-forming miRNases are high.

The study of the cleaving activity of the developed BCs allowed us to determine the main regularities and parameters of efficient miRNA cleavage by engineered miRNases. The obtained data indicate that the overall architecture of the miRNA molecule, including the sequence of the bulge loop and adjacent motifs, strongly affects the reactivity of phosphodiester bonds in the bulge and the efficiency of RNA cleavage by miRNase. Indeed, the miRNA-specific conjugates from similar structural categories (either α- or β-conformers) and with the same or similar hybridization powers may exhibit very different cleavage potencies, which seem to be driven by the sequence of the bulge-loop region. For example, miR-17-specific miRNases generally show high potency, presumably because they cleave RNA predominantly at pyrimidine–A bonds, which are known to be sensitive to cleavage in bulge loops [48]. By contrast, miR-21, with the predominance of purine–purine linkages in the central part of its sequence, appeared to be resistant to cleavage. This suggests that purine-rich bulge loops are less favourable for degradation by miRNases than those formed from pyrimidine–A motifs. The obtained data show that the conjugates are able to cleave any bond in the induced bulge loop; however, the central phosphodiester bonds in the three-nucleotide bulges, namely, the second and third bonds, seem to respond better to cleavage by BCs.

Moreover, A–T and A–U base pairs flanking the bulge were estimated to facilitate cleavage because they had partially drifted apart at the single-strand–double-strand junction, providing additional freedom. Indeed, in the case of miR-21, the cleavage of the bulge, induced by miR-21–BC-α1/β1, flanked with A–U base pairs at both sides, was more profound than that of the bulge induced by miR-21–BC-α2/β2 neighboring with G–C base pairs (Figure 3f). In the case of miR-17, the two A–U base pairs adjacent to the induced bulge loop from the 5′ side likewise facilitated the cleavage of the first bond in the bulge U10–A11 (Figure 3c).

The previously established synergism of the combined action of miRNase and RNase H [23,25,28] is a key prerequisite for the therapeutic efficiency of the conjugates in cells. Earlier, it was revealed using molecular dynamics simulations that, regardless of the α- or β-configuration of the peptides, the complex of the bulge-forming conjugate and RNA adopts the intermediate conformation between the A and the B forms of the helix. Although the induced bulge loop leads to some degree of helical bending, the overall structure does not experience any significant conformational change [38], which can potentially be recognized with natural enzymes (e.g., RNase H). The design of the conjugates was carried out in such a way that the length of the RNA–DNA duplex from the 5′ and 3′ sides of the peptide attachment site was at least 8 nt, which was expected to be sufficient for the recognition and binding of RNase H, followed by the endonucleolitic cleavage of miRNA. In this study, we have provided experimental evidence that the proposed structural design of BC–miRNases does not affect the substrate properties of the heteroduplex formed by BCs and miRNA, and RNase H was able to recognize RNA–DNA hybrid regions and perform cleavage with high efficiency.

The pattern of RNase-H-induced miRNA cleavage in the heteroduplex with an oligonucleotide or a conjugate (i.e., cleavage at the G19–G20, G7–C8, and H6–G7 bonds) indicates that RNase H indeed perceives the heteroduplex as two separate 5′ and 3′ heteroduplexes separated by the bulge loop. Thus, each miRNA–BC–miRNase heteroduplex presumably attracts two RNase H molecules, each of which cleaves the miRNA in one of its functionally significant determinants—the seed region and the terminal region. As a result of the joint action of BC–miRNase and RNase H, the miRNA molecule is cut in three regions—the central loop by the conjugate and the seed and terminal regions by RNase H, which, in addition to irreversible breaks, leads to a complete destabilization of the duplex and an increase in the release rate of miRNase and RNase H for subsequent cycles of cleavage of the next copies of miRNA. The core result of the study is the identification of the synergism of the joint action of BC–miRNase and RNase H as early as the first hour; a significant increase in the rate of miRNA cleavage by both BC–miRNase and RNase H was observed. The recruitment of two nucleases possessing the autonomous multiple-turnover ability is an invaluable advantage of the developed strategy for efficient multiple rounds of scission of numerous copies of RNA and increased silencing activity in cells.

**Figure 7:**
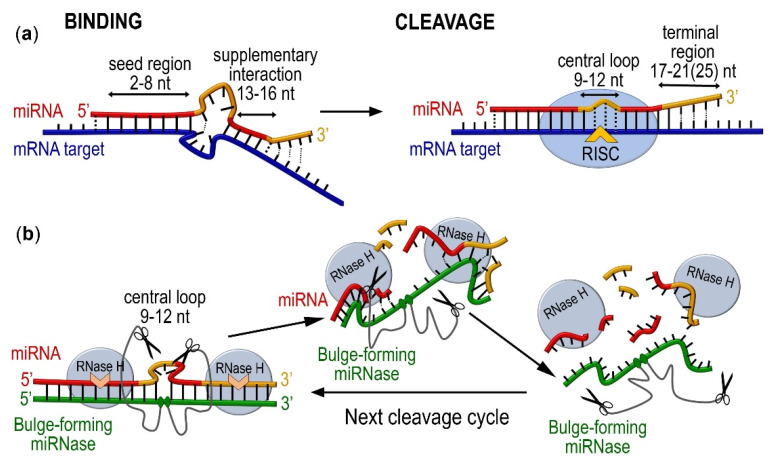
Functionally significant regions in the miRNA molecule which can be used as target sites for catalytic destruction by a combination of bulge-forming miRNase and RNase H. (**a**) Cellular mechanism of miRNA-guided mRNA repression through binding with the miRNA seed region (2–8 nt) reinforced by hybridization with the supplementary interaction region (13–16 nt), followed by RISC-mediated cleavage at the mRNA target site aligned with the miRNA central loop (9–13 nt). (**b**) Proposed strategy for knockdown miRNA through synergetic cleavage by BC–miRNase and RNase H, when the cleavage at the central loop of miRNA is catalysed by BC–miRNase, while the concurrent degradation of the seed region and terminal region is catalysed by RNase H, which is abundant in cells.

The miRNA region chosen for cleavage by miRNases deserves special attention. Summarizing currently available data, four functionally significant determinants were distinguished in miRNA sequences [52,53,54] that are critical for their biological action (see Figure 7a), which are: (i) the seed region (2–8 nt), (ii) the central loop (9–13 nt), (iii) the region of supplementary pairing (13–16 nt), and (iv) the terminal region (17–21/17–23 nt). The key stages of miRNA-induced mRNA repression include the binding and cleavage of a target mRNA sequence. It is broadly accepted that the seed region is the primary determinant for targeting efficacy and sequence specificity [52,53,54]. However, miRNA–target affinity and specificity are often reinforced by the miRNA supplementary pairing region, which contributes to an improvement in target recognition, redistributes miRNA affinity and specificity between different mRNA targets, and enhances the repression of seed-matched target mRNA. The seed region, with the assistance of the supplementary pairing region, controls the selection for miRNA–mRNA binding [52,54]. The miRNA central loop was found to be critical for the establishment of a cleavage-competent conformation and it is required for efficient target cleavage, mediated by RISC. In fact, mismatches in the 9–11 positions of miRNA lead to significant reductions in or even the failure of target cleavage [52]. The terminal region, in addition to its involvement in target RNA-directed microRNA degradation (TDMD), also controls target mRNA degradation. A decrease in the level of binding in the terminal region promotes an increase in the rate of the multiple turnover of mRNA target cleavage [52].

Novel bulge-forming miRNases were targeted to the miRNA central loop (Figure 7b). The degradation of this region by BC–miRNases undoubtedly rendered the miRNA completely incompetent, nullified mRNA cleavage, and, as a consequence, resulted in a loss of its correct functioning in the cell. The obtained data show that RNase H activity can not only result in a manifold increase in the rate of miRNA cleavage by the BC–miRNase in the central loop but also entirely degrade the miRNA molecule due to RNase-H-mediated cleavage in the seed and terminal regions (Figure 7b).

To conclude, this study represents the first example of the application of bulge-forming peptide–oligonucleotide conjugates for the degradation of oncogenic miRNA sequences in a functionally important part of the molecule—the central loop. The synergistic recruitment of the intracellular activity of RNase H may potentially increase the efficiency of the degradation of pathogenic miRNA due to the parallel destruction of the seed region and the terminal region of the miRNA to achieve the desired therapeutic effect.

## 4. Materials and Methods

### 4.1. Oligonucleotides, Peptides, and Reagents

Oligodeoxyribonucleotides incorporating an internal abasic nucleotide with an aminohexyl linker attached at the C1′ position in an α- or a β-configuration were synthesized in the Laboratory of Medicinal Chemistry, Institute of Chemical Biology and Fundamental Medicine (Novosibirsk, Russia), according to the standard phosphoramidite protocol in an ASM-800 synthesizer (Biosset, Novosibirsk, Russia) using a solid support, nucleoside phosphoramidites, and a chemical phosphorylation reagent from Glenn Research (Sterling, VA, USA). α- and β-aminohexyl abasic sugar phosphoramidite monomers were purchased from Link Technologies Ltd. (Bellshill, Scotland, UK). Oligonucleotides were isolated using consecutive ion-exchange (Polysil SA-500 columns, Russia) and reverse-phase HPLC (LiChrosorb RP-18 columns, Merck, Kenilworth, NJ, USA) according to the standard protocols. The sequences of the oligonucleotides are given in Figure 1. Acetyl-[LRLRG]_2_-CO_2_H was purchased from Biomatik (Kitchener, ON, Canada). The reagents and materials were purchased from Sigma-Aldrich (Burlington, MA, USA), unless otherwise indicated. Water was purified in-house using a Milli-Q purification system (Millipore, Burlington, MA, USA).

### 4.2. miR-17–miRNase and miR-21–miRNase Conjugate Synthesis

MiR-17–miRNase and miR-21–miRNase conjugates were synthesized using amide coupling chemistry following a protocol described previously [38,39]. The reaction was carried out through the conjugation of the peptide Acetyl-[LRLRG]_2_-CO_2_H, pre-activated with 4-dimethylaminopyridine (DMAP) and N, N’-dicyclohexylcarbodiimide (DCC), to the oligonucleotide component, which was converted into DMSO-soluble cetyltrimethylammonium bromide (CTAB) salt prior to the reaction. The conjugates were isolated from the reaction mixtures by precipitation with 4% LiClO4 (*w*/*v*) in acetone and purified using RP-HPLC and a semi-preparative Phenomenex Luna C-18 column, as described previously [38,39]. The identity and purity of all conjugates were confirmed using MALDI–ToF/ToF spectrometry (see Figures S2, S4, and S6) and ^1^H NMR spectroscopy (see Figures S3, S5, and S7). In all cases, oligonucleotide concentrations were estimated using a UV–Vis spectrophotometer (Varian Cary 4000 dual beam, Santa Clara, CA, USA) at a 260 nm wavelength using molar extinction coefficients. A Bruker Daltonics Ultraflex TOF/TOF mass spectrometer (Bruker, Billerica, MA, USA) was used to carry out a mass spectrometry analysis of the conjugates using the time-of-flight matrix-assisted laser desorption ionisation (MALDI) technique.

**miR-17–BC-α**. MALDI-MS: *m*/*z* = 7588.0 [M + Li]^+^ adduct MW = 7581.0 g mol^−1^ calcd. (Figure S2a).

Hydrogen-1 NMR (Figure S3a) (D_2_O with TSP (0.01 mM), 400 MHz): δ 0.72–0.80 (m, 24H, Leu- H^δ^), 0.90–2.95 (m, 100H, 21 × H2′ and 21×H2′’ sugar ring protons, 15H (5 × CH_3_ of 5 × dT), 8H of 8 × Arg-H^β^, 8H of 8 × Arg-H^γ^, 8H of 8 × Leu-H^β^, 4H of 4 × Leu-H^γ^, 12H of 6 × CH^2^ (aminohexyl linker), 3H of acetyl-CH_3_), 3.18 (m, 8H, 8 × Arg-H^δ^), 3.42–4.40 (m, 75H, 63 × H4′/H5′H5” sugar ring protons, 4H of 2 × Gly-CH_2_, 8H of 8 × Leu/Arg-Hα), 5.50–6.35 (m, 28H, 21 × H1′ sugar ring protons, 7 × H5 of dC), 7.21–8.41 (m, 25H, 25 × Ar-H from dG(H8×3), dA(H8 × 5), dA(H2 × 5), dC(H6 × 7), and dT(H6 × 5)). H3′ sugar ring protons (4.3–5.2 ppm) were not analyzed due to the suppression of the residual water signal at 4.76 ppm. Not all H4′/H5′H5” sugar ring protons were fully resolved, and the suppression of residual water prevented signal detection.

**miR-17–BC-β**. MALDI-MS: *m*/*z* = 7589.0 [M + Li]^+^ adduct MW = 7581.0 g mol^−1^ calcd. (Figure S2b).

Hydrogen-1 NMR (Figure S3b) (D_2_O with TSP (0.01 mM), 400 MHz): δ 0.72–0.80 (m, 24H, Leu- H^δ^), 0.90–2.95 (m, 100H, 21 × H2′ and 21 × H2′’ sugar ring protons, 15H (5 × CH_3_ of 5 × dT), 8H of 8 × Arg-H^β^, 8H of 8 × Arg-H^γ^, 8H of 8 × Leu-H^β^, 4H of 4 × Leu-H^γ^, 12H of 6 × CH_2_ (aminohexyl linker), 3H of acetyl-CH_3_), 3.18 (m, 8H, 8 × Arg-H^δ^), 3.42–4.40 (m, 75H, 63 × H4′/H5′H5” sugar ring protons, 4H of 2 × Gly-CH_2_, 8H of 8 × Leu/Arg-Hα), 5.50–6.35 (m, 28H, 21 × H1′ sugar ring protons, 7 × H5 of dC), 7.21–8.41 (m, 25H, 25 × Ar-H from dG(H8 × 3), dA(H8 × 5), dA(H2 × 5), dC(H6 × 7), and dT(H6 × 5)). H3′ sugar ring protons (4.3–5.2 ppm) were not analyzed due to the suppression of the residual water signal at 4.76 ppm. Not all H4′/H5′H5” sugar ring protons were fully resolved, and the suppression of residual water prevented signal detection.

**miR-17–BC-αα**. MALDI-MS: *m*/*z* = 9102.23 MW = 9103.85 g mol^−1^ calcd. (Figure S4a).

Hydrogen-1 NMR (Figure S5a) (D_2_O with TSP (0.01 mM), 400 MHz): δ 0.72–0.80 (m, 48H, Leu- H^δ^), 0.90–2.95 (m, 145H, 22 × H2′ and 22 × H2′’ sugar ring protons, 15H (5 × CH_3_ of 5 × dT), 16H of 16 × Arg-H^β^, 16H of 16 × Arg-H^γ^, 16H of 16 × Leu-H^β^, 8H of 8 × Leu-H^γ^, 24H of 2 × (6 × CH_2_) (2 × aminohexyl linkers), 6H of 2 × acetyl-CH_3_), 3.18 (m, 16H, 16 × Arg-H^δ^), 3.42–4.40 (m, 90H, 66 × H4′/H5′H5” sugar ring protons, 8H of 4 × Gly-CH_2_, 16H of 16 × Leu/Arg-Hα), 5.50–6.35 (m, 29H, 22 × H1′ sugar ring protons, 7 × H5 of dC), 7.21–8.41 (m, 25H, 25 × Ar-H from dG(H8 × 3), dA(H8 × 5), dA(H2 × 5), dC(H6 × 7), and dT(H6 × 5)). H3′ sugar ring protons (4.3–5.2 ppm) were not analyzed due to the suppression of the residual water signal at 4.76 ppm. Not all H4′/H5′H5” sugar ring protons were fully resolved, and the suppression of residual water prevented signal detection.

**miR-17–BC-ββ**. MALDI-MS: *m*/*z* = 9105.91 [M + H]^+^ MW = 9103.85 g mol^−1^ calcd. (Figure S4b).

Hydrogen-1 NMR (Figure S5b) (D_2_O with TSP (0.01 mM), 400 MHz): δ 0.72–0.80 (m, 48H, Leu- H^δ^), 0.90–2.95 (m, 145H, 22 × H2′ and 22 × H2′’ sugar ring protons, 15H (5 × CH_3_ of 5 × dT), 16H of 16 × Arg-H^β^, 16H of 16 × Arg-H^γ^, 16H of 16 × Leu-H^β^, 8H of 8 × Leu-H^γ^, 24H of 2 ×(6 × CH_2_) (2 × aminohexyl linkers), 6H of 2 × acetyl-CH_3_), 3.18 (m, 16H, 16 × Arg-H^δ^), 3.42–4.40 (m, 90H, 66 × H4′/H5′H5” sugar ring protons, 8H of 4 × Gly-CH_2_, 16H of 16 × Leu/Arg-Hα), 5.50–6.35 (m, 29H, 22 × H1′ sugar ring protons, 7 × H5 of dC), 7.21–8.41 (m, 25H, 25 × Ar-H from dG(H8 × 3), dA(H8 × 5), dA(H2 × 5), dC(H6 × 7), and dT(H6 × 5)). H3′ sugar ring protons (4.3–5.2 ppm) were not analyzed due to the suppression of the residual water signal at 4.76 ppm. Not all H4′/H5′H5” sugar ring protons were fully resolved, and the suppression of residual water prevented signal detection.

**miR-21–BC-α1.** MALDI-MS: *m*/*z* = 7333.1 [M + K]^+^ adduct MW = 7294.6 g mol^−1^ calcd. for [C_250_H_354_N_95_O_126_P_19_] (Figure S6a).

Hydrogen-1 NMR (Figure S7a) (D_2_O with TSP (0.01 mM), 400 MHz): δ 0.69–0.77 (m, 24H, Leu- H^δ^), 1.10–2.78 (m, 98H, 20 × H2′ and 20 × H2′’ sugar ring protons, 15H (5 × CH_3_ of 5 × dT), 8H of 8 × Arg-H^β^, 8H of 8 × Arg-H^γ^, 8H of 8 × Leu-H^β^, 4H of 4 × Leu-H^γ^, 12H of 6 × CH_2_ (aminohexyl linker), 3H of Acetyl-CH_3_), 3.1 (m, 8H, 8 × Arg-H^δ^), 3.36–4.37 (m, 72H, 60 × H4′/H5′H5” sugar ring protons, 4H of 2 × Gly-CH_2_, 8 × Leu/Arg-H^α^), 5.36–6.38 (m, 24H, 20 × H1′ sugar ring protons, 4 × H5 of dC), 7.17–8.38 (m, 27H, 27 × Ar-H from dG(H8 × 2), dA(H8 × 8), dA(H2 × 8), dC(H6 × 4), and dT(H6 × 5)). H3′ sugar ring protons (4.3–5.2 ppm) were not analyzed due to the suppression of the residual water signal at 4.76 ppm. Not all H4′/H5′H5” sugar ring protons were fully resolved, and the suppression of residual water prevented signal detection.

**miR-21–BC-β1.** MALDI-MS: *m*/*z* = 7333.1 [M + K]^+^ adduct MW = 7294.6 g mol^−1^ calcd. For [C_250_H_354_N_95_O_126_P_19_] (Figure S6b).

Hydrogen-1 NMR (Figure S7b) (D_2_O with TSP (0.01 mM), 400 MHz): δ 0.69–0.77 (m, 24H, Leu- H^δ^), 0.99–2.77 (m, 98H, 20 × H2′ and 20 × H2′’ sugar ring protons, 15H (5 × CH_3_ of 5 × dT), 8H of 8 × Arg-H^β^, 8H of 8 × Arg-H^γ^, 8H of 8 × Leu-H^β^, 4H of 4 × Leu-H^γ^, 12H of 6 × CH_2_ (aminohexyl linker), 3H of Acetyl-CH_3_), 3.1 (m, 8H, 8 × Arg-H^δ^), 3.30–4.38 (m, 72H, 60 × H4′/H5′H5” sugar ring protons, 4H of 2 × Gly-CH_2_, 8 × Leu/Arg-Hα), 4.92–6.28 (m, 24H, 20 × H1′ sugar ring protons, 4 × H5 of dC), 7.18–8.30 (m, 27H, 27 × Ar-H from dG(H8 × 2), dA(H8 × 8), dA(H2 × 8), dC(H6 × 4), and dT(H6 × 5)). H3′ sugar ring protons (4.3–5.2 ppm) were not analyzed due to the suppression of the residual water signal at 4.76 ppm. Not all H4′/H5′H5” sugar ring protons were fully resolved, and the suppression of residual water prevented signal detection.

**miR-21–BC-α2.** MALDI-MS: *m*/*z* = 7358.2 [M + K]^+^ adduct MW = 7319.6 g mol^−1^ calcd. for [C_250_H_353_N_98_O_125_P_19_] (Figure S6c).

Hydrogen-1 NMR (Figure S7c) (D_2_O with TSP (0.01 mM), 400 MHz): δ 0.70–0.78 (m, 24H, Leu- H^δ^), 0.95–2.95(m, 95H, 20 × H2′ and 20 × H2′’ sugar ring protons, 12H (4 × CH_3_ of 4 × dT), 8H of 8 × Arg-H^β^, 8H of 8 × Arg-H^γ^, 8H of 8 × Leu-H^β^, 4H of 4 × Leu-H^γ^, 12H of 6 × CH_2_ (aminohexyl linker), 3H of Acetyl-CH_3_), 3.08 (m, 8H, 8 × Arg-H^δ^), 3.29–4.35 (m, 72H, 60 × H4′/H5′H5” sugar ring protons, 4H of 2 × Gly-CH_2_, 8 × Leu/Arg-H^α^), 5.32–6.33 (m, 24H, 20 × H1′ sugar ring protons, 4 × H5 of dC), 7.11–8.38 (m, 27H, 27 × Ar-H from dG(H8 × 3), dA(H8 × 8), dA(H2 × 8), dC(H6 × 4), and dT(H6 × 4)). H3′ sugar ring protons (4.3–5.2 ppm) were not analyzed due to the suppression of the residual water signal at 4.76 ppm. Not all H4′/H5′H5” sugar ring protons were fully resolved, and the suppression of residual water prevented signal detection.

**miR-21–BC-β2.** MALDI-MS: *m*/*z* = 7358.3 [M + Kl]^+^ adduct MW = 7319.6 g mol^−1^ calcd. for [C_250_H_353_N_98_O_125_P_19_] (Figure S6d).

Hydrogen-1 NMR (Figure S7d) (D_2_O with TSP (0.01 mM), 400 MHz): δ 0.71–0.79 (m, 24H, Leu- H^δ^), 1.14–2.90 (m, 95H, 20 × H2′ and 20 × H2′’ sugar ring protons, 12H (4 × CH_3_ of 4 × dT), 8H of 8 × Arg-H^β^, 8H of 8 × Arg-H^γ^, 8H of 8 × Leu-H^β^, 4H of 4 × Leu-H^γ^, 12H of 6 × CH_2_ (aminohexyl linker), 3H of Acetyl-CH_3_), 3.09 (m, 8H, 8 × Arg-H^δ^), 3.29–4.39 (m, 72H, 60 × H4′/H5′H5” sugar ring protons, 2 × Gly-CH_2_, 8 × Leu/Arg-H^α^), 5.47–6.34 (m, 24H, 20 × H1′ sugar ring protons, 4 × H5 of dC), 7.12–8.38 (m, 27H, 27 × Ar-H from dG(H8 × 3), dA(H8 × 8), dA(H2 × 8), dC(H6 × 4), and dT(H6 × 4)). H3′ sugar ring protons (4.3–5.2 ppm) were not analyzed due to the suppression of the residual water signal at 4.76 ppm. Not all H4′/H5′H5” sugar ring protons were fully resolved, and the suppression of residual water prevented signal detection.

### 4.3. RNA Labelling

The 5′-end labelling using [^32^P]–ATP and T4 polynucleotide kinase (Thermo Scientific, Waltham, MA, USA) and the isolation of synthetic single-stranded [^32^P]-miRNAs miR-21–5p 5′-UAGCUUAUCAGACUGAUGUUGA-3′ and miR-17–5p 5′-CAAAGUGCUUACAGUGCAGGUAG-3′ were carried out according to a procedure previously described [55,56].

### 4.4. Gel-Retardation Assay

The reaction mixture (4 µL) containing 100 cpm (Cherenkov’s counting) of [^32^P]-miRNA, 1 μM unlabelled miRNA, a conjugate at a concentration ranging from 0.1 to 10 μM and one of the buffers: Buffer 1, 50 mM Tris–HCl, pH 7.0, 200 mM KCl, and 1 mM EDTA or Buffer 2, 20 mM Tris–HCl, pH 7.8, 40 mM KCl, 8 mM MgCl_2_, and 1 mM DTT, was incubated at 37 °C for 45 min and quenched by the adding of a loading buffer (20% ficoll, 0.025% bromophenol blue, 0.025% xylene cyanol). The samples were loaded onto the running gel immediately after quenching the reaction, with 1 min intervals. The formation of the complex miRNA–conjugate was analyzed using electrophoresis in 15% native PAAG at 4 °C. The gels were analyzed using Molecular Imager FX. The extent of the binding of the conjugate to miRNA was determined as the ratio of the radioactivity measured in the complex to the total radioactivity applied onto the gel lane. The association constants (Ka) were estimated as follows:

(1) $$K_{a}=\frac{\alpha }{{\left[\mathrm{BC}\right]}_{0}\;\left(1-\alpha \right)\;\left(1-\alpha \;\left(\frac{{\left[\mathrm{miR}\right]}_{0}}{{\left[\mathrm{BC}\right]}_{0}}\right)\right)}$$

where α is the fraction of bound RNA and [miR]_0_ and [BC]_0_ are the concentrations of miRNA and the conjugate, respectively.

### 4.5. Ribonuclease Activity Assay

The reaction mixture (10 µL) contained 800 cpm (Cherenkov’s counting) of [^32^P]-miRNA, 1 μM unlabelled miRNA, one of the conjugates at a concentration 20 μM, and one of the buffers: Buffer 1, 50 mM Tris–HCl, pH 7.0, 200 mM KCl, and 1 mM EDTA or Buffer 2, 20 mM Tris–HCl, pH 7.8, 40 mM KCl, 8 mM MgCl_2_, and 1 mM DTT. The mixture was incubated at 37 °C for 72 h. The aliquots were quenched by the precipitation of RNA with 2% LiClO_4_ in acetone (90 µL). RNA was collected using centrifugation and dissolved in loading buffer (8 M urea, 0.025% bromophenol blue, 0.025% xylene cyanol). RNA cleavage products were analyzed in 18% PAAG/8M urea using TBE (100 mM Tris-borate, pH 8.3, 2 mM EDTA) as running buffer. To identify the cleavage sites, an imidazole and T1-ladders produced by partial RNA cleavage with 2 M imidazole buffer (pH 7.0) [57] and with RNase T1 [58], respectively, were run in parallel. To obtain quantitative data, the gels were dried and analyzed using Molecular Imager FX. The data were analyzed using the Quantity One software. The total extent of RNA cleavage was determined as the ratio of the total intensity of the bands related to the miRNA cleavage products to the total intensity of all fragments, including uncleaved miRNA. The extent of RNA cleavage at an individual site was determined as the ratio of the intensity of the separate band to the total intensity of all fragments, including uncleaved miRNA. The observed rate constants (k_obs_) of conjugate-mediated miRNA cleavage were deduced from the following exponential equation:

(2) $$F\left(t\right)=\alpha \times \left(1-e^{-k_{obs\;}\cdot t}\right)$$

where *t* is time, *α* is the substrate concentration, and *F*(*t*) is the fraction of the substrate cleaved at time *t*.

### 4.6. Ribonuclease Activity Assay in the Presence of RNase H

The reaction mixture (10 µL) containing 800 cpm (Cherenkov’s counting) of [^32^P]-miRNA, 1 µM unlabelled miRNA, 20 µM either of BC or bulge-forming oligonucleotides (B-ON) in Buffer 2, 20 mM Tris–HCl, pH 7.8, 40 mM KCl, 8 mM MgCl_2_, and 1 mM DTT was incubated at 37 °C for 20 min. RNase H (Thermo Fisher Scientific, Waltham, MA, USA) at a concentration of 5 u/mL was then added. The mixtures were further incubated at 37 °C for 72 h. The aliquots were quenched and the RNA cleavage products were collected and analyzed as described above.

## Figures and Tables

**Table 1:** The main hybridization and cleavage properties of bulge-loop-forming miRNases.

Conjugates *	Molecular Mass Calculated/Observed	Ka, ×10^6^, M^−1^	Total Cleavage, 72 h, %	k_obs_, ×10^−6^, s^−1^
**miR-21-BC-α1**	7294.6/7333.1 (+ K^+^)	**96.0** ± 30.0 ^(1)^ **26.9** ± 8.5 ^(2)^	**8.3** ± 1.3 ^(2)^	**0.38** ± 0.03 ^(2)^
**miR-21-BC-α2**	7319.6/7358.3 (+ K^+^)	n.d.	**7.1** ± 0.9 ^(2)^	**0.28** ± 0.01 ^(2)^
**miR-21-BC-β1**	7294.6/7333.1 (+ K^+^)	**4.9** ± 1.7 ^(1)^ **0.8** ± 0.3 ^(2)^	**19.5** ± 1.9 ^(2)^	**0.87** ± 0.05 ^(2)^
**miR-21-BC-β2**	7319.6/7358.3 (+ K^+^)	n.d.	**13.3** ± 1.2 ^(2)^	**0.57** ± 0.03 ^(2)^
**miR-17-BC-α**	7581.0/7588.0 (+ Li^+^)	**46.1** ± 15.4 ^(1)^ **3.6** ± 1.2 ^(2)^	**27.2** ± 3.1 ^(1)^ **29.1** ± 2.0 ^(2)^	**1.31** ± 0.08 ^(1)^ **1.48** ± 0.12 ^(2)^
**miR-17-BC-β**	7581.0/7589.0 (+ Li^+^)	**42.7** ± 13.4 ^(1)^ **4.5** ± 1.4 ^(2)^	**23.2** ± 2.6 ^(1)^ **25.5** ± 1.8 ^(2)^	**1.13** ± 0.06 ^(1)^ **1.19** ± 0.03 ^(2)^
**miR-17-BC-αα**	9103.85/9102.23	n.d.	**57.1** ± 4.5 ^(2)^	**4.86** ± 0.27 ^(2)^
**miR-17-BC-ββ**	9103.85/9105.91	n.d.	**94.9** ± 5.6 ^(2)^	**13.9** ± 3.26 ^(2)^

* The structures of the conjugates are shown in Figure 1. ^(1)^ Buffer 1: 50 mM Tris–HCl, pH 7.0, 200 mM KCl and 1 mM EDTA. ^(2)^ Buffer 2: 20 mM Tris–HCl, pH 7.8, 40 mM KCl, 8 mM MgCl_2_, and 1 mM DTT. n.d.: not determined.

## Data Availability

Not applicable.

## Supplementary Materials

The following supporting information can be downloaded at: https://www.mdpi.com/article/10.3390/ijms23126562/s1.
