# Supplementary material for: Bulge-Forming miRNases Cleave Oncogenic miRNAs at the Central Loop Region in a Sequence-Specific Manner
Source: Int J Mol Sci. 2022 Jun 12;23(12):6562. doi: 10.3390/ijms23126562 (PMC9224474; doi:10.3390/ijms23126562)
Supplement: Supplementary file 1 [file ijms-23-06562-s001.zip › SUPPLEMENTARY MATERIALS.pdf]

## Supplementary materials

# Bulge-forming miRNases cleave oncogenic miRNAs at the central loop region in a sequence-specific manner

**Olga Patutina [1,†], Daria Chiglintseva [1,†], Bahareh Amirloo [2], David Clarke [2], Svetlana Gaponova [1], Valentin Vlassov [1], Elena Bichenkova [2,‡], and Marina Zenkova [1,‡,*]**

[1] Institute of Chemical Biology and Fundamental Medicine SB RAS, Lavrentiev's ave. 8, 630090, Novosibirsk, Russia; patutina@niboch.nsc.ru (O.P.); dashachiglintseva@gmail.com (D.Ch.); sveta-mira@yandex.ru (S.G.); vvv@niboch.nsc.ru (V.V.)

[2] University of Manchester, Faculty of Biology, Medicine and Health, School of Health Sciences, Oxford Rd, Manchester, M13 9PT, UK; elena.v.bichenkova@manchester.ac.uk (E.B.); bahareh.amirloo@manchester.ac.uk (B.A.); david.clarke@manchester.ac.uk (D.C.)

* Correspondence: marzen@niboch.nsc.ru; Tel. +7-383-3635160 or +7-9059376611 (M.Z.)

† These authors contributed equally to this work as first authors

‡ These authors contributed equally to this work as lead authors

**Table of contents:**

1. Predicted complexes between miR-17 or miR-21 sequences and various recognition oligonucleotides with full or partial complementarity……2
2. MALDI-TOF spectra of the mono-conjugates miR-17–BC-α and miR-17–BC-β……3
3. $^{1}$H NMR spectra of the mono-conjugates miR-17–BC-α and miR-17–BC-β……4
4. MALDI-TOF spectra of the bis-conjugates miR-17–BC-αα and miR-17–BC-ββ……5
5. $^{1}$H NMR spectra of the bis-conjugates miR-17–BC-αα and miR-17–BC-ββ……6
6. MALDI-TOF spectra of the mono-conjugates miR-21–BC-α1, miR-21–BC-β1, miR-21–BC-α2 and miR-21–BC-β2……7
7. $^{1}$H NMR spectra of the mono-conjugates miR-21–BC-α1, miR-21–BC-β1, miR-21–BC-α2 and miR-21–BC-β2……8
8. Hybridization of [$^{32}$P]-miRNAs with BCs in Buffer 2……9
9. Self-cleavage of 5'-[$^{32}$P]-miR-21 and miR-17 in a complex with the bulge-forming nonconjugated oligonucleotides miR-21–B-ON and miR-17–B-ON in the presence of $Mg^{2+}$ ions……10
10. The most stable hairpins and homodimers of miR-21 and miR-17 according to OligoAnalyzer™ Tool analysis……11

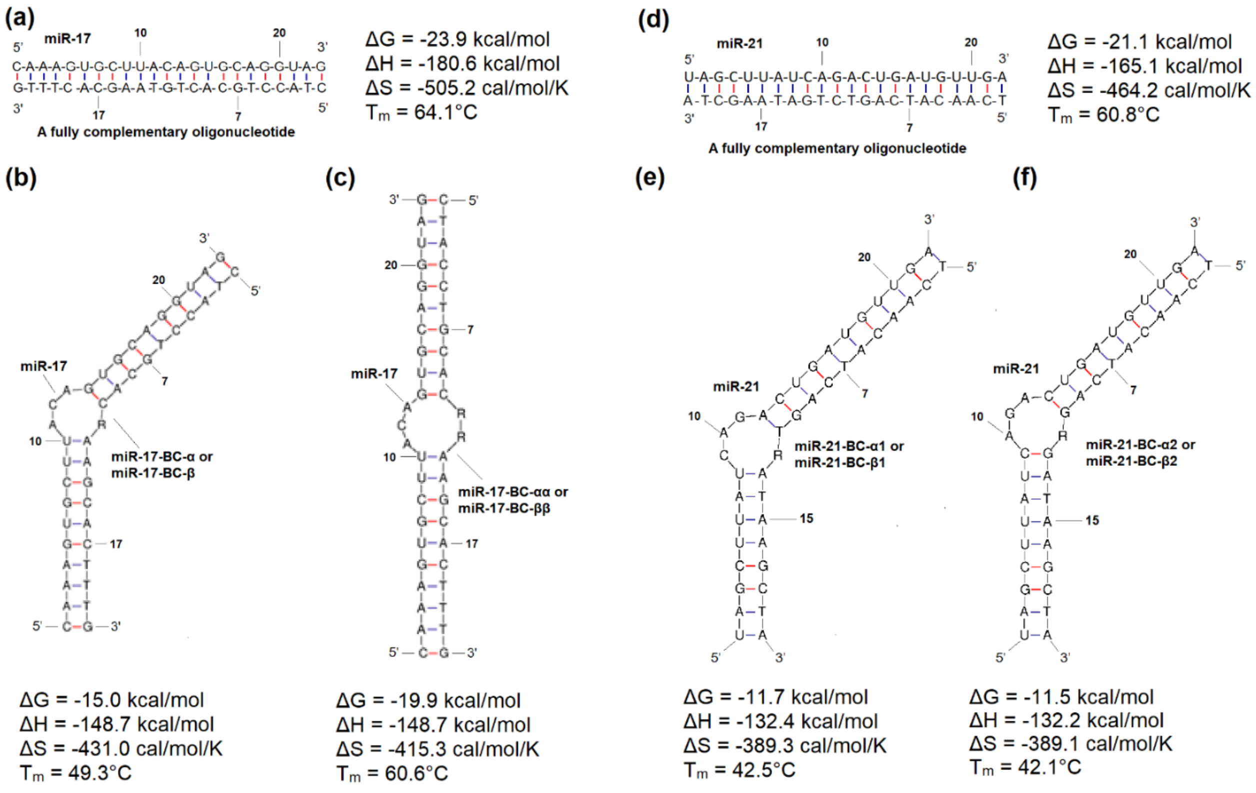


**Figure S1**. Predicted complexes between miR-17 (**a**)-(**c**) or miR-21 (**d**)-(**f**) sequences and various recognition oligonucleotides with full or partial complementarity. The secondary structures and corresponding thermodynamics parameters (ΔG, ΔH, ΔS and Tm) were calculated using the DINAMelt Server (www.unafold.org).

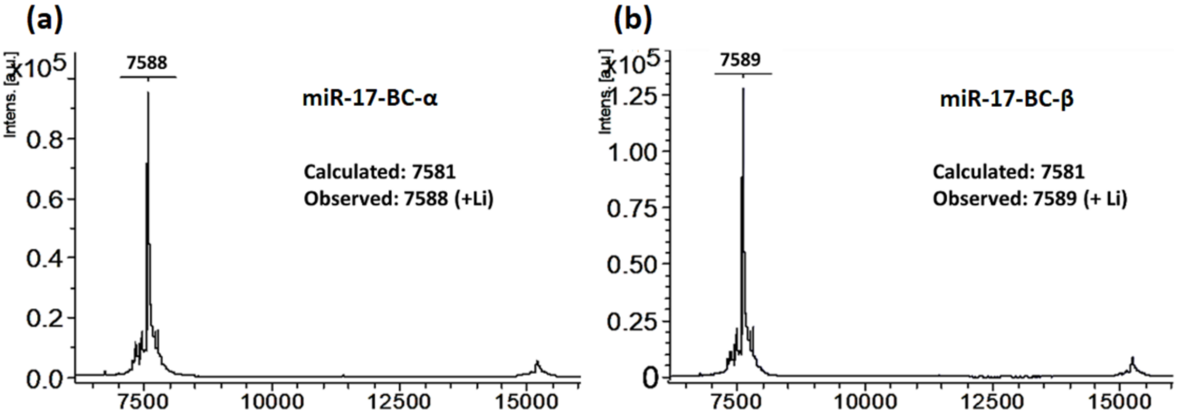


**Figure S2.** MALDI-TOF spectra of the mono-conjugates miR-17–BC-α (**a**) and miR-17–BC-β (**b**). The spectra were recorded using a 0.7 M 3-hydroxy picolinic acid matrix (97 mg/mL, with 0.07 M ammonium citrate, 16 mg/mL in 50:50 ACN: $H_2O$) on a Bruker Daltonics Ultraflex ToF/ToF mass spectrometer, using the positive ion detection mode.

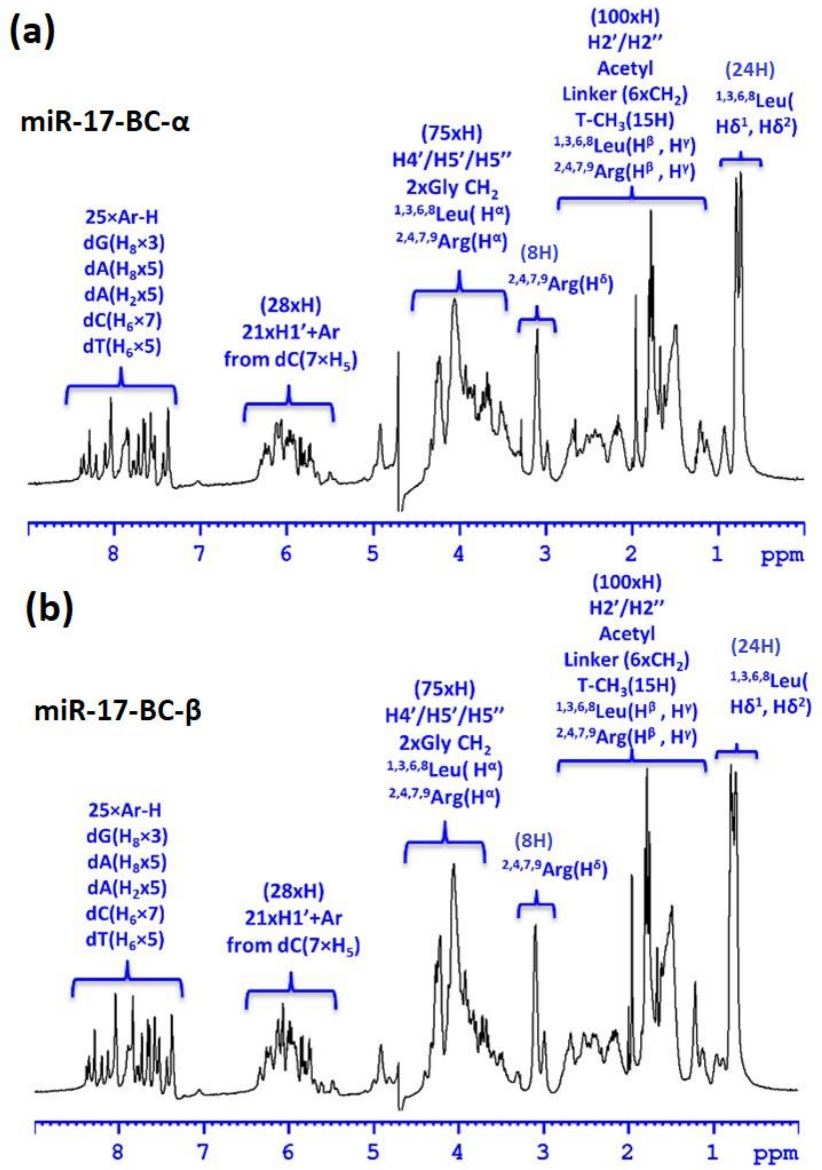


**Figure S3.** $^1$H NMR spectra (400 MHz, Bruker Avance II 400) of the mono-conjugates miR-17–BC-*α* (**a**) and miR-17–BC-β (**b**) were isolated from the reaction mixture using RP-HPLC, indicating prominent a chemical shift of the protons from the oligonucleotides, peptide, aminohexyl linker and acetyl protecting group. In each spectrum, the breakdown of proton assignment for each region as well as the integral intensity was indicated. The H3' region was assigned since water suppression prohibited a full assignment.

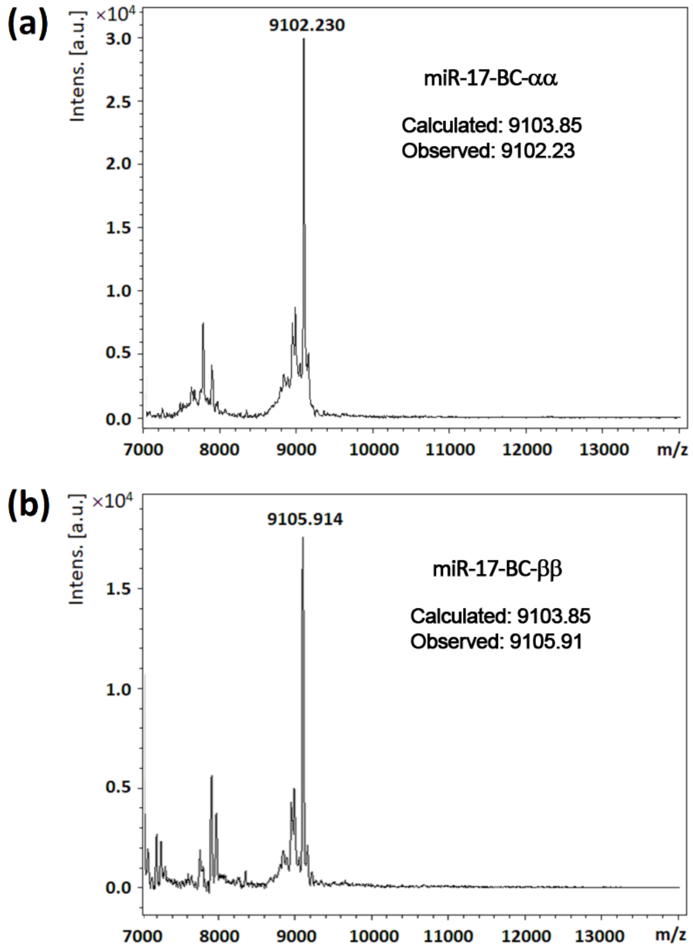


**Figure S4.** MALDI-TOF spectra of the bis-conjugates miR-17–BC-αα (**a**) and miR-17–BC-ββ (**b**). The spectra were recorded using the THAP (2,4,6-trihydroxyacetophenone monohydride) (25 mg/mL) matrix, with the addition of ammonium citrate (5 mg/mL) in a 50:50 ratio of ACN: $H_2O$ (1:1) and 0.1% TFA) on a Bruker Daltonics Autoflex Speed MALDI-TOF mass spectrometer, using the positive ion detection mode.

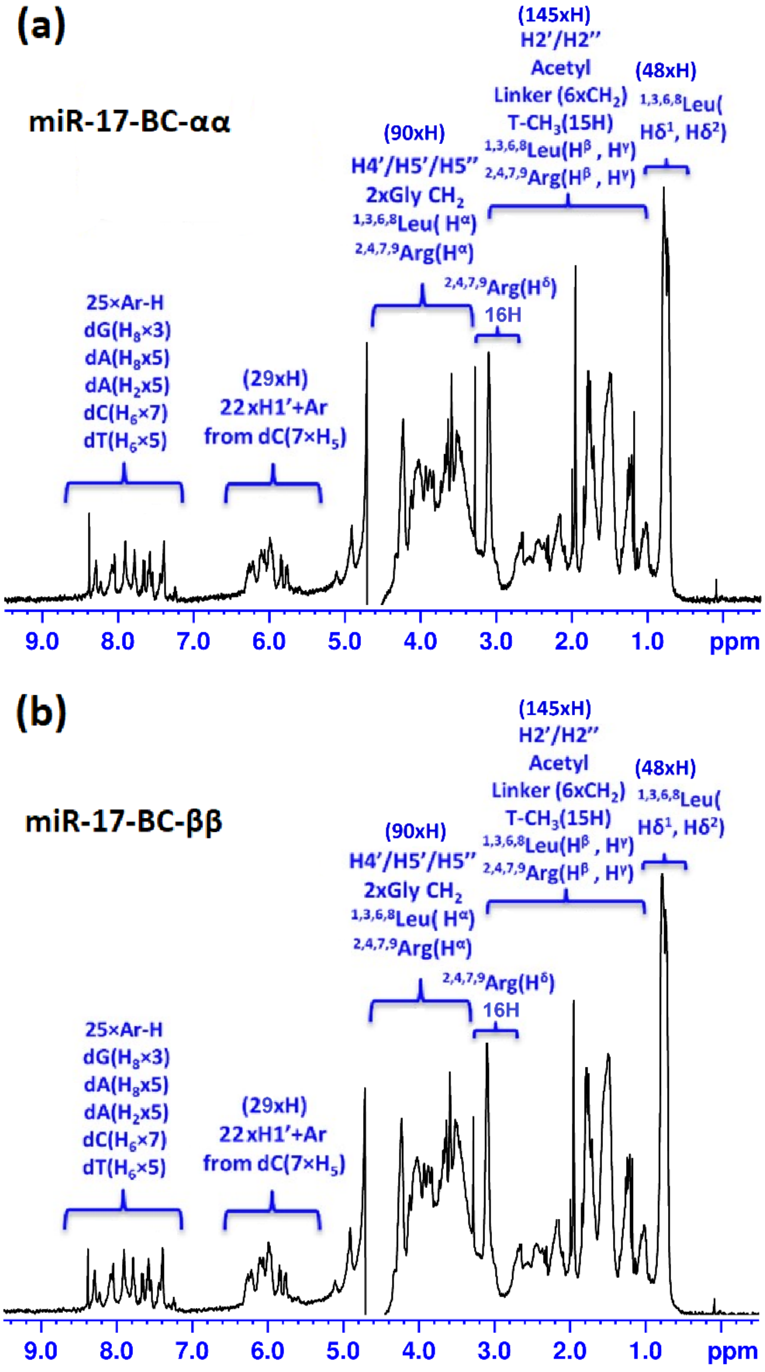


**Figure S5.** $^1$H NMR spectra (400 MHz, Bruker Avance II 400) of the bis-conjugates miR-17–BC-*αα* (**a**) and miR-17–BC-ββ (**b**) were isolated from the reaction mixture using RP-HPLC, indicating a prominent chemical shift of protons from the oligonucleotides, peptide, aminohexyl linker and acetyl protecting group. In each spectrum, the breakdown of proton assignment for each region as well as the integral intensity was indicated. The H3′ region was not assigned since water suppression prohibited a full assignment.

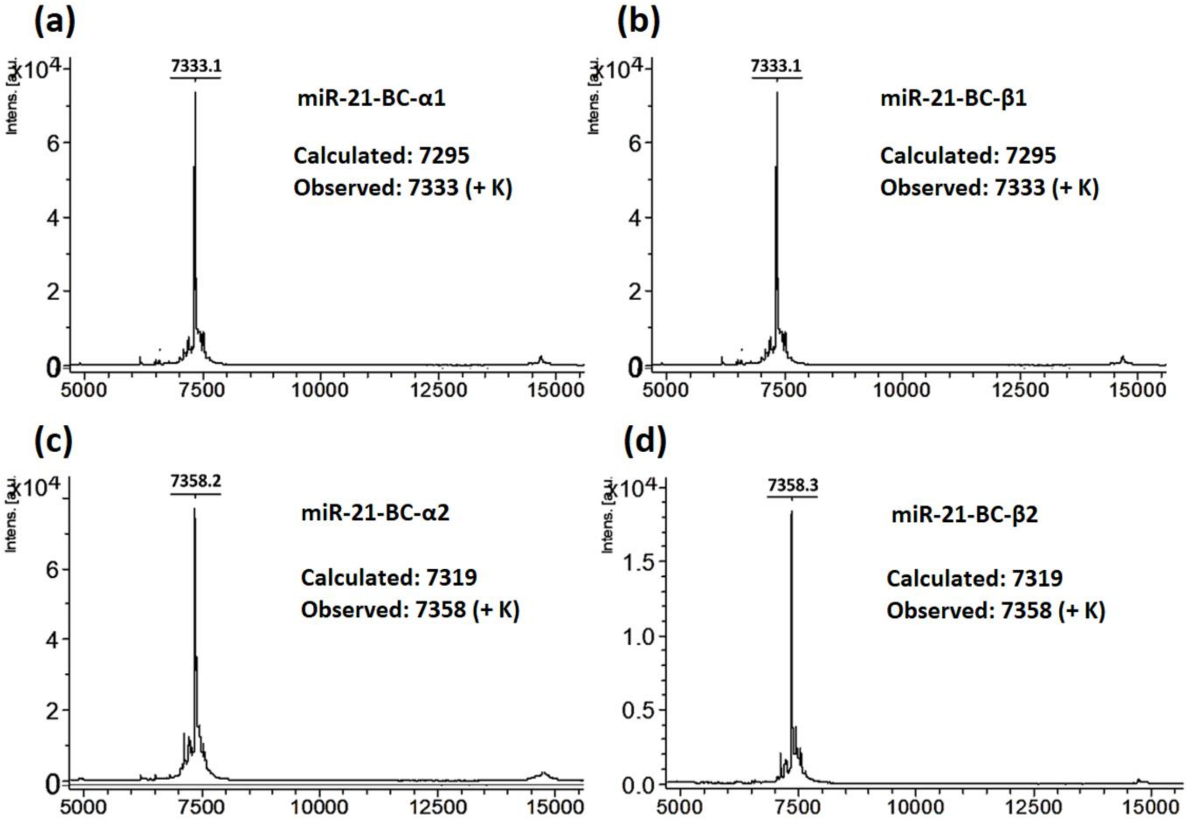


**Figure S6.** MALDI-TOF spectra of the mono-conjugates miR-21–BC-$\alpha$1 (**a**), miR-21–BC-β1 (**b**), miR-21–BC-$\alpha$2 (**c**) and miR-21–BC-β2 (**d**). The spectra were recorded using a 0.7 M 3-hydroxy picolinic acid matrix (97 mg/mL, with 0.07 M ammonium citrate, 16 mg/mL in a 50:50 ratio of ACN: $H_2O$) on a Bruker Daltonics Ultraflex ToF/ToF mass spectrometer using the positive ion detection mode.

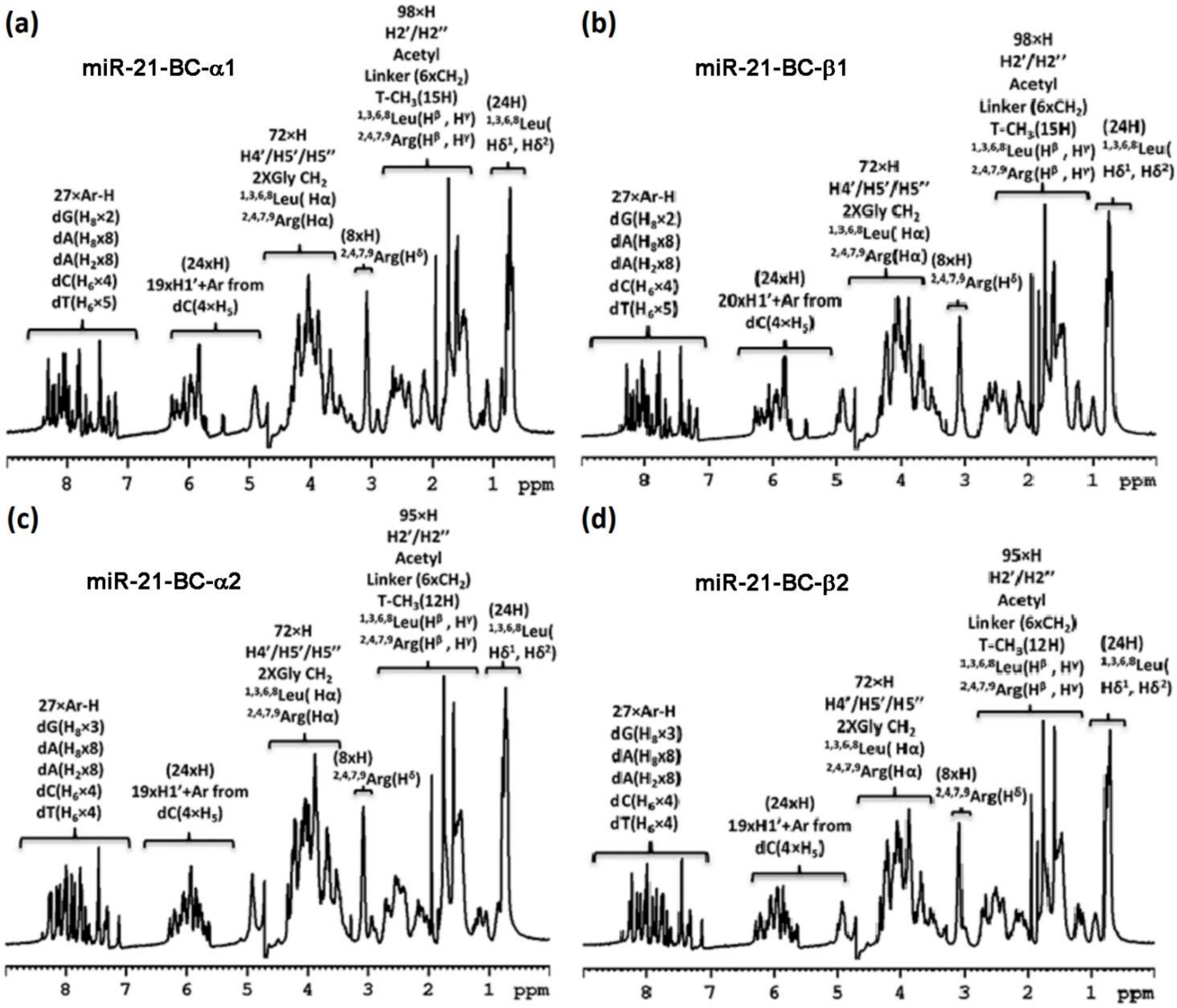


**Figure S7.** $^1$H NMR spectra (400 MHz, Bruker Avance II 400) of the mono-conjugates miR-21–BC-$\alpha$1 (**a**), miR-21–BC-$\beta$1 (**b**), miR-21–BC-$\alpha$2 (**c**) and miR-21–BC-$\beta$2 (**d**), showing the characteristic resonance areas of the oligonucleotide protons, peptide protons, aminohexyl linker and acetyl protecting group. The assignments of the key $^1$H resonance regions are indicated above each spectrum. A 1:1 stoichiometric ratio of peptide to oligonucleotide was confirmed through integration. The spectra were recorded in $D_2O$ at 25 °C.

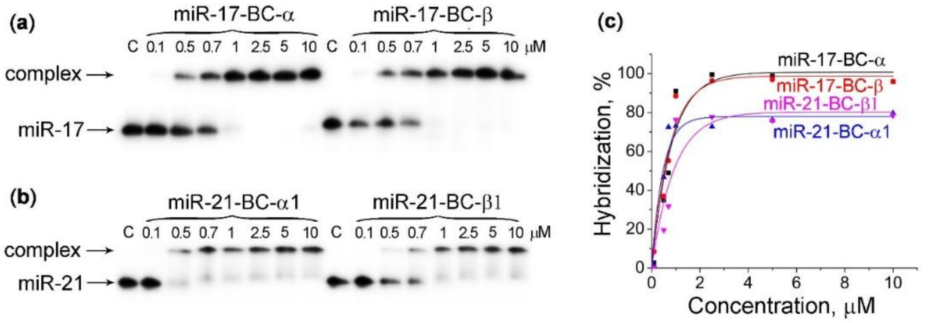


**Figure S8.** Hybridization of [$^{32}$P]-miRNAs with BCs in Buffer 2. (**a**, **b**) Radioautographs of 15% native PAAG, showing hybridization of miR-17–BC-α and miR-17–BC-β with miR-17 and miR-21–BC-α1, miR-21–BC-β1 with miR-21, respectively. The miRNAs (1 μM) were incubated with BCs (0.1–10 μM) in Buffer 2 (20 mM Tris-HCl, pH 7.8, 40 mM KCl, 8 mM $MgCl_2$ and 1 mM DTT) at 37 °C for 45 min. C—miRNAs were incubated in the same buffer in the absence of conjugates. The samples were loaded onto the running gel immediately after the reaction was quenched, with 1 min intervals. The concentration (μM) of the conjugate is indicated on the top of electropherograms. (**c**) Concentration profiles of BC hybridization efficiency with miR-17 and miR-21.

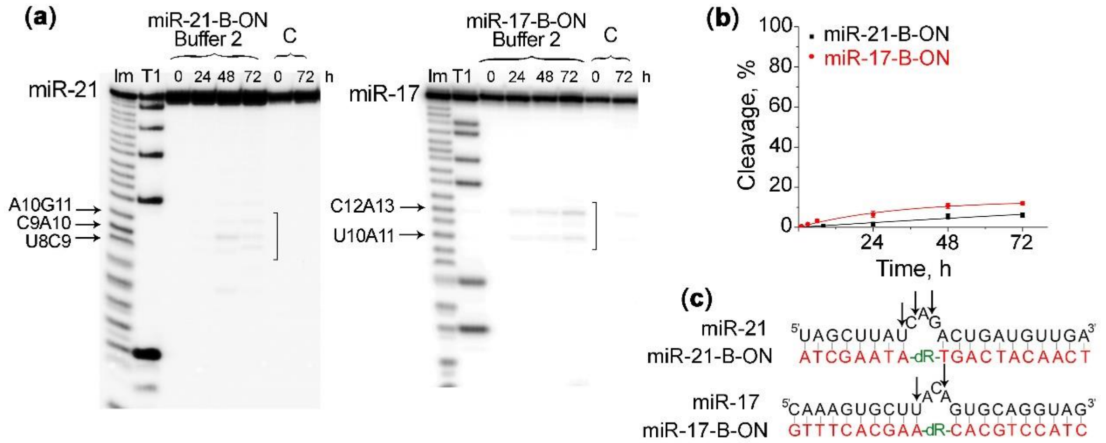


**Figure S9.** Self-cleavage of 5'-[$^{32}$P]-miR-21 and miR-17 in a complex with the bulge-forming nonconjugated oligonucleotides miR-21–B-ON and miR-17–B-ON in the presence of $Mg^{2+}$ ions (Buffer 2). (**a**) Radioautographs of 18% denaturing PAAG showing the cleavage products of miR-21 and miR-17 in Buffer 2. The miRNA-21 (1 μM) and oligonucleotides (20 μM) were incubated at 37°C for 72 h. Lanes Im and T1—imidazole ladder and partial RNA digestion with RNase T1, respectively; C—miRNAs were incubated in the same buffer in the absence of oligonucleotides. The incubation time is shown at the top. The square bracket indicates the bulge-loop region. (**b**) Progress curves of miR-21 and miR-17 self-cleavage in the complex with oligonucleotides in Buffer 2. (**c**) Positions of miR-21 and miR-17 self-cleavage in a complex with miR-21–B-ON and miR-17–B-ON, respectively.

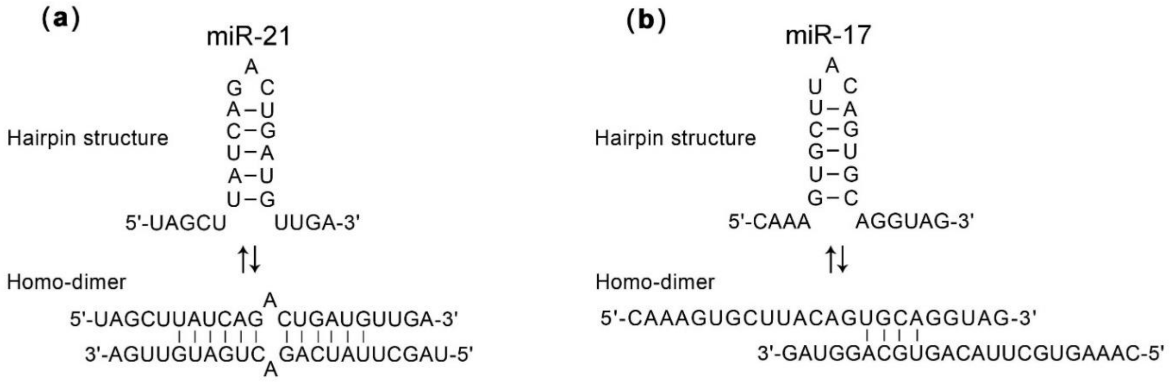


**Figure S10.** The most stable hairpins and homodimers of miR-21 (**a**) and miR-17 (**b**) according to OligoAnalyzer™ Tool analysis.
